# Identification of variant HIV envelope proteins with enhanced affinities for precursors to anti-gp41 broadly neutralizing antibodies

**DOI:** 10.1371/journal.pone.0221550

**Published:** 2019-09-10

**Authors:** Hong Zhu, Elizabeth Mathew, Sara M. Connelly, Jeffrey Zuber, Mark Sullivan, Michael S. Piepenbrink, James J. Kobie, Mark E. Dumont

**Affiliations:** 1 Department of Biochemistry and Biophysics, University of Rochester Medical Center, Rochester, NY, United States of America; 2 Department of Microbiology and Immunology, University of Rochester Medical Center, Rochester, NY, United States of America; 3 Infectious Diseases Division, University of Rochester Medical Center, Rochester, NY United States of America; New York State Department of Health, UNITED STATES

## Abstract

HIV envelope protein (Env) is the sole target of broadly neutralizing antibodies (BNAbs) that are capable of neutralizing diverse strains of HIV. While BNAbs develop spontaneously in a subset of HIV-infected patients, efforts to design an envelope protein-based immunogen to elicit broadly neutralizing antibody responses have so far been unsuccessful. It is hypothesized that a primary barrier to eliciting BNAbs is the fact that HIV envelope proteins bind poorly to the germline-encoded unmutated common ancestor (UCA) precursors to BNAbs. To identify variant forms of Env with increased affinities for the UCA forms of BNAbs 4E10 and 10E8, which target the Membrane Proximal External Region (MPER) of Env, libraries of randomly mutated Env variants were expressed in a yeast surface display system and screened using fluorescence activated cell sorting for cells displaying variants with enhanced abilities to bind the UCA antibodies. Based on analyses of individual clones obtained from the screen and on next-generation sequencing of sorted libraries, distinct but partially overlapping sets of amino acid substitutions conferring enhanced UCA antibody binding were identified. These were particularly enriched in substitutions of arginine for highly conserved tryptophan residues. The UCA-binding variants also generally exhibited enhanced binding to the mature forms of anti-MPER antibodies. Mapping of the identified substitutions into available structures of Env suggest that they may act by destabilizing both the initial pre-fusion conformation and the six-helix bundle involved in fusion of the viral and cell membranes, as well as providing new or expanded epitopes with increased accessibility for the UCA antibodies.

## Introduction

Despite the success of antiretroviral drugs for controlling HIV infection, and their potential for preventing the spread of HIV infection, there remains an urgent need for a vaccine that is capable of preventing infection. Availability of a vaccine with even modest efficacy would be an effective and economically accessible route for ending the HIV pandemic [1]. However, the considerable effort invested to date in developing an anti-HIV vaccine has yet to result in a useful immunogen. Despite more than a hundred initial clinical trials, only six formulations have entered efficacy trials, of which only one showed detectable protection; the Thai RV144 trial, which yielded a tentative 31% efficacy [2, 3].

Efforts to develop a vaccine capable of eliciting antibody-mediated immune protection against HIV have focused on the HIV envelope glycoprotein (Env), the major surface glycoprotein on the virus that is the target of all known neutralizing anti-HIV antibodies. Much of the difficulty of developing a vaccine stems from the high rate of mutation of Env, since most immunodominant epitopes are readily mutated, allowing the virus to escape binding and neutralization by antibodies. Nonetheless, a subset of infected individuals develop antibodies to HIV that are capable of neutralizing a broad range of viruses [4], though these responses usually occur too slowly to protect those individuals. Such Broadly Neutralizing Antibodies (BNAbs) have generally been found to target epitopes on the HIV envelope protein that cannot be altered without diminishing function of the protein, such as its roles in binding receptors and fusing viral and cellular membranes. The existence of naturally occurring BNAbs suggests that it should be possible to develop an HIV vaccine by identifying an immunogen capable of eliciting these responses.

Env is initially expressed as a gp160 form that undergoes cleavage by cellular furin proteases into gp140 and gp41 fragments, which remain associated with each other. The Membrane Proximal External region (MPER) of gp41 is a major target of BNAbs, including some of the most broadly protective antibodies identified to date [5–7]. This region of Env is also considered to be a promising target for BNAbs because of its relative lack of sequence diversity. However, there have, so far, been only limited attempts to identify immunogens capable of eliciting broadly neutralizing anti-MPER responses [8–11].

Most mature BNAbs that have been identified in HIV-infected individuals exhibit high rates of somatic hypermutation resulting in sequences that are highly diverged from their inferred germline precursors. The rates of somatic mutation for anti-HIV BNAbs are generally much higher than the rates for antibodies to pathogens other than HIV [12–15]. Furthermore, reconstructed germline Unmutated Common Ancestor (UCA) forms of anti-HIV BNAbs generally fail to bind or bind only weakly to HIV Env [12, 16–20]. Since binding of antigens to antibody precursors displayed as receptors on the surface of B cells is required to initiate the program of antibody maturation, the failure of normal forms of Env to bind to the UCA forms of BNAbs appears to be a significant barrier to the elicitation of broadly neutralizing immune responses and may be an evolutionary strategy that the HIV virus uses to evade attack by the immune system. Thus, the identification of variant forms of Env with enhanced affinities for binding UCA forms of BNAbs seems a plausible strategy for developing immunogens likely to elicit broadly neutralizing anti-HIV responses. This has previously been attempted by various means, including computational “rational” design of scaffolded epitopes derived from Env [6, 7, 21–24], generation of peptides corresponding to regions of Env that serve as epitopes of BNAbs [25], removal of glycosylation sites from Env to promote surface exposure of effective epitopes [11, 26], high-order multimerization of epitopes derived from Env [24], and screens of libraries of randomly mutated forms of Env to identify variants exhibiting enhanced affinities for UCAs and partially somatically mutated precursors to BNAbs [21–24, 27–29].

We report here the use of a yeast surface display system in conjunction with a library of randomly mutated forms of Env to screen for variants with enhanced affinities for reconstructed UCAs for two anti-MPER BNAbs, 4E10[30] and 10E8[5–7]. Such screening has the dual advantages of maximizing affinity of binding of Env variants to the BNAb precursors while simultaneously identifying variants in which the relevant epitopes are maximally accessible for antibody binding at the molecular surface of Env. There are also important advantages to screening for immunogens in a system that is outside the context of normal viral expression of Env: Screening in the yeast system allows exploration of a wider range of sequence space of Env variants than would be possible in the context of a viable virus since variant forms of Env that bind to BNAb UCAs do not need to retain their functions in viral infection and propagation in order to elicit the desired immune responses. Furthermore, evolutionary pressures during viral infection and propagation that may constrain the diversity of Env sequences in order to minimize recognition by components of the immune system are not operative in the heterologous display system.

We have identified distinct, but partially overlapping, sets of Env variants that exhibit enhanced affinities for binding to the 4E10 and 10E8 UCAs. The recovered mutations did not reside in the known epitopes for the mature versions of the two antibodies, although the epitopes for the mature BNAbs appear to contribute to the binding of the variants to the UCA forms of the antibodies. The mutations occurred at positions in the Env sequence that would be likely to diminish viability and propagation if they were altered in the context of an intact virus. They appear to generate new epitopes and promote exposure of epitopes for anti-MPER antibodies by destabilizing characterized conformations of Env.

## Results

### Construction and screening of mutational libraries

To identify variant forms of Env with enhanced affinities for UCA precursors to MPER-targeted BNAbs, we chose starting forms of Env that could be well-expressed in the yeast surface display system and exhibited robust binding to mature anti-MPER antibodies. These consisted of gp140 constructs containing the full complement of variable loop regions as well as mutations previously found to enhance expression and binding to mature forms of BNAbs [31] that we will refer to as “dsm” (designed SOSIP with stabilizing mutations) forms of Env (sequences shown in S1 Fig). These constructs include the SOSIP disulfide linking the gp120 and gp41 portions of Env [32], as well as a peptide cleavage site between the gp120 and gp41 moieties that was optimized for cleavage by the yeast furin protease Kex2p [33]. The constructs were displayed at the yeast cell surface by fusing their C-termini to a V5 epitope and to Aga2p, a yeast agglutinin that attaches via disulfide bonds to the Aga1p protein that is part of the yeast cell wall [34] (S2 Fig).

**Fig 1:**
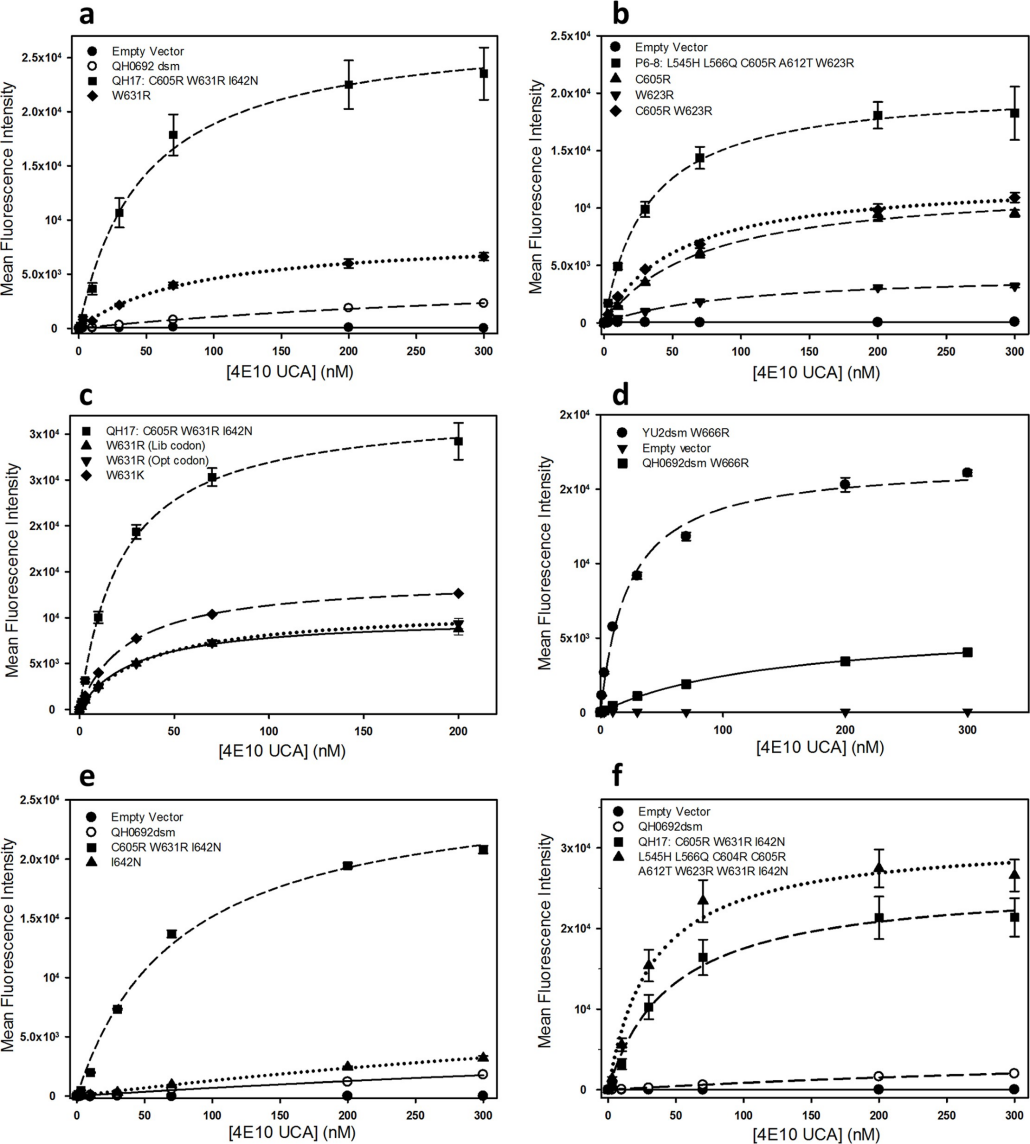
a) Saturation binding of 4E10 UCA to reconstructed QH0692dsm Env variants related to sorted clone QH-17. b) Saturation binding of 4E10 UCA to reconstructed QH0692dsm Env variants related to sorted clone P6-8. c) Saturation binding of 4E10 UCA to reconstructed QH0692dsm Env variants carrying substitutions at position W631. d) Saturation binding of 4E10 UCA to reconstructed QH0692dsm and YU2dsm Env variants carrying substitutions at position W666. e) Saturation binding of 4E10 UCA to QH0692dsm Env variants carrying substitutions at position I642. f) Saturation binding of 4E10 UCA to QH0692dsm Env variants carrying combined mutations.

**Fig 2:**
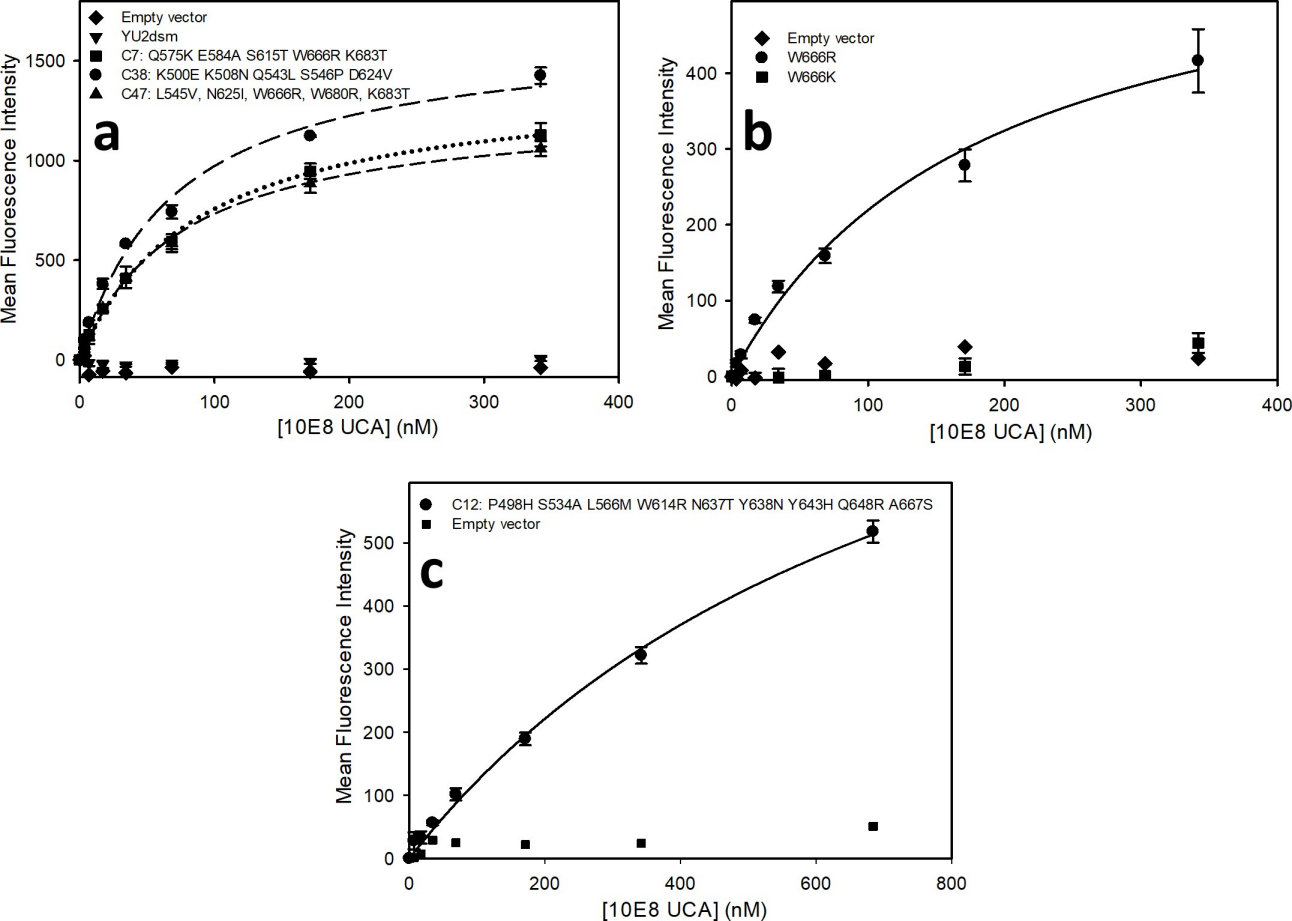
a) Saturation binding assay of 10E8 UCA to reconstructed YU2dsm Env variants corresponding to clones C7, C38, and C47 from 10E8 UCA-sorted library (containing the amino acid substitutions noted on figure). b) Saturation binding assay of 10E8 UCA to reconstructed YU2dsm Env variants carrying mutations W666R and W666K to 10E8 UCA. c) Saturation binding assay of 10E8 UCA to reconstructed library clone C12 lacking W666R but containing the substitutions P498H, S534A, L566M, W614R, N637T, Y638N, Y643H, Q648R, and A667S.

The starting form of Env used for screening for variants that bind to the UCA of 4E10 was the gp140dsm form of Env from the QH0692 strain of HIV, which had previously been shown to exhibit weak binding to some UCA forms of BNAbs [35]. The 4E10 UCA bound only weakly to yeast-displayed QH0692dsm gp140 (Fig 1)[33]. However, mutagenesis of Env derived from strain QH0692 was not optimal for screening for 10E8 UCA-binding variants because the gp140dsm form of QH0692 Env does not bind well even to the mature form of 10E8 [33]. Thus, to screen for Env variants that bind to the 10E8 UCA, an additional mutational library was constructed based on the gp140dsm Env from the YU2 strain of HIV, which showed robust binding to mature 10E8 [33] but no detectable binding to the 10E8 UCA (Fig 2).

Mutagenesis was performed by PCR using an error-prone polymerase under two different sets of conditions that yielded mean nucleotide mutation rates of 0.62% and 0.65% for the QH0692 and YU2 libraries, respectively, providing a mean number of mutations of 2.7 and 3.3 over the mutagenized regions (see Methods). PCR products were co-transformed into yeast with gapped plasmid, yielding libraries of ~2 × 10^7^ transformants in which each transformant was derived from a different PCR product (S2 Fig). Multiple rounds of two-color Fluorescence Activated Cell Sorting (FACS) screening with UCA forms of the anti-Env antibodies and with anti-V5 epitope binding were used to enrich for cells expressing forms of Env with enhanced affinity for the UCA antibodies (S3 Fig). The use of two-color screening reduced the background of cells that map to the gated region even in cells displaying non-mutagenized receptors. The sorting process is outlined in Table 1. Slight enrichment of a population of cells exhibiting elevated binding to the 4E10 UCA (compared to cells displaying un-mutagenized QH0692dsm Env) was observed after one round of sorting but no detectable enrichment for binding of 10E8 UCA to YU2dsm Env was detected until after two rounds of sorting (S3 Fig).

**Table 1:** Mutagenic screens for UCA-binding Env variants.

	Number of clones	
Screening step	QH0692 library (4E10)	YU2 library (10E8)
Size of library	2 × 10^7^	1 × 10^7^
Clones FACS sorted (1^st^ sort)	3 × 10^7^	4 × 10^7^
Clones collected (1^st^ sort)^a^	4 × 10^4^	3 × 10^5^
Clones FACS sorted 2^nd^ sort)	5 × 10^5^	6 × 10^6^
Clones collected (2^nd^ sort)^a^	1 × 10^4^	3 × 10^4^
Clones FACS sorted 3^rd^ sort)	-	1 × 10^6^
Clones collected (3^rd^ sort)^a^	-	5 × 10^3^
Clones tested	40	48

^a^corrected for ~50% cell viability following sorting and regrowth.

### Mutations in QH0692 Env that confer binding to the 4E10 UCA

In individual single-point binding assays of yeast clones selected after two rounds of sorting for 4E10 UCA binding, 28 of 32 tested clones exhibited more than 2-fold greater mean fluorescence than that of the starting QH0692 gp140dsm form of Env (S1 Table). Sequencing of these 28 clones revealed a variety of mutations dominated by the substitution W631R, which was recovered in 17 clones, W623R (recovered in 5 clones), and C605R (recovered in 5 clones) (S1 Table). The sequenced clones also contained numerous additional mutations demonstrating that they were derived from independent transformants from the library. The distribution of sequences obtained from Next Generation Sequencing (NGS) of the population of cells following one round of sorting for 4E10 UCA binding confirmed the enrichment for W631R (present in 61% of reads), C605R (present in 11% of reads), W623R (present in 5% of reads), as well as revealing a surprising enrichment for additional Trp to Arg substitutions including W666R (present in two individual sorted clones and 3% of NGS reads), and W610R (present in two individual sorted clones and 2% of NGS reads). Eight of the ten most abundant amino acids changes among the NGS reads from the sorted cells were Trp to Arg substitutions (Table 2).

**Table 2:** Most abundant mutations recovered in NGS analysis of screen for binding to 4E10 UCA.

	Frequency of Recovery		
Amino acid Substitution	Overall^a^	Presence of W631R^b^	Absence of W631R^c^
W631R	0.605	1.000	0
C605R	0.113	0.026	0.247
W623R	0.049	0.030	0.078
W666R	0.029	0.022	0.083
Q619R	0.027	0.015	0.045
W610R	0.021	0.009	0.040
W628R	0.017	0.006	0.034
Y638H	0.017	0.014	0.021
L646S	0.017	0.014	0.021
W614R	0.017	0.022	0.008
L568Q	0.016	0.013	0.021
G572D	0.016	0.013	0.021
W670R	0.015	0.009	0.025
L669S	0.015	0.016	0.012
I603N	0.015	0.010	0.021
Q590H	0.014	0.011	0.018
W571R	0.013	0.012	0.015
L568P	0.013	0.003	0.027
C605S	0.012	0.007	0.019
M626K	0.012	0.015	0.006
S615T	0.011	0.012	0.008
Q552L	0.011	0.006	0.018
L555M	0.010	0.013	0.007
N640D	0.010	0.011	0.009
V580D	0.010	0.005	0.018
I635K	0.010	0.0002	0.024

^a^The listed substitutions include substitutions recovered in the sorted library at a frequency of >1% from a total of 560,076 reads of sequences that appeared more than once in the NGS of the 4E10 UCA-sorted library. (All the mutations shown occurred at frequencies more than 3 standard deviations above the mean frequency of substitution of any particular nucleotide substitution in the unsorted library which was: 0.0021 ± 0.0017)

^b^from a total of 339,040 reads of sequences containing W631R that appeared more than once in the NGS of the 4E10 UCA-sorted library.

^c^from the total of 221,036‬ reads of sequences lacking W631R that appeared more than once in the NGS of the 4E10 UCA-sorted library.

Clones from the sorted library that exhibited the largest fluorescence changes in assays of 4E10 UCA binding were re-created by site-directed mutagenesis and their binding to 4E10 UCA was examined using saturation binding experiments, monitored by flow cytometry. Several of the re-created Env variants bound 4E10 UCA with at least 10-fold higher apparent affinity compared to the unmutagenized QH0692dsm Env. The cells displaying these variants also appeared to have a greater number of surface binding sites compared to cells expressing the unmutagenized form. (Fig 1A and 1B and Table 3), though this could, at least in part, be explained by a slower off-rate in the non-equilibrium binding assays. These increases in binding of Env variants to the 4E10 UCA are not the result of overall increased expression levels of the surface-displayed Env, as the maximal levels of binding of anti-V5 epitope antibody to cells expressing the sorted variants were generally within 2-fold of the levels for binding to the un-mutagenized QH0692dsm Env (S4A Fig). Also, none of the variants exhibited any increase in the ability to bind to an irrelevant IgG, daclizumab (DAC), a humanized anti-interleukin 2 receptor CD25 antibody [36](S11 Fig).

**Table 3:** Binding parameters for mutation combinations reconstructed in the QH0692 Env by site-directed mutagenesis.

Mutations	Clone name	4E10 UCA binding	
		Relative K_D_^a^	Relative B_max_^a^
QH0692dsm starting form of Env	**-**	18 ± 1	0.3 ± 0.04
W631R	**-**	1.8 ± 0.3	0.3 ± 0.1
W631K	**-**	1.2 ± 0.1	0.4 ± 0.1
C605R	**-**	3.3 ± 0.1	0.4 ± 0.1
W623R	**-**	3.1 ± 0.3	0.2 ± 0.1
W666R	**-**	3.2 ± 0.1	-^b^
I642N	**-**	14 ± 5	0.6 ± 0.2
C605R W631R	**-**	1.2 ± 0.1	0.6 ± 0.1
C605R W623R	**-**	1.7 ± 0.1	0.6 ± 0.1
C605R W631R I642N	QH-17	1	1
L545H L566Q C605R A612T W623R	P6-8	1.4 ± 0.1	0.5 ± 0.04
L545H, L566Q, C604S, C605R, A612T, W623R, W631R, I642N	Combined	0.7 ± 0.1	1.2 ± 0.1

^a^K_D_ and B_max_ values are reported relative to the the QH-17 variant form of Env assayed in parallel with the indicated variant forms. The overall K_D_ for QH-17 from the different assays was 44 ± 3 nM (n = 24).

^b^Not measured

The high affinity of 4E10 UCA binding to Env variants containing the most frequently-recovered substitution, W631R, is primarily due to the introduction of the positively charged side chain at this position. An Env variant containing the single amino acid substitution W631R binds to the 4E10 UCA with an apparent K_D_ only ~1.8-fold higher than the K_D_ for binding of the tightest binding multiply substituted library clone (QH-17) from the sorted library (Fig 1C, Table 3). Furthermore, substitution of lysine at position 631 had effects similar to substitution of arginine (Fig 1C, Table 3). The significance of substituting a charged residue for W631 is further supported by the identification of W631K mutations at a frequency of 0.4% among total reads in the sorted 4E10 UCA-binding library and at a frequency of 1% among the reads that do not contain the W631R mutation, despite the fact that the W631K substitution requires a double base TGG→AAG substitution that is expected to occur at random at a frequency of only 0.0008% (calculated as the product of the frequencies in the unsorted library of the T→A substitution in the first position of the codon (0.4%) and G→A in the second position (0.2%)).

The amino acid substitutions C605R, W623R, and W666R that were recovered in the individual sorted clones and at elevated frequencies among the NGS reads of the 4E10 UCA-sorted library, when tested as individual amino acid changes, also resulted in enhancement of apparent affinities and numbers of sites compared to the starting form of QH0692dsm Env (Fig 1, Table 3). In contrast to what was observed at position W631, no substitutions of Lys for C605, W623, or W666 were seen among the sorted NGS reads (though substitution of lysine for C605 would require an unlikely triple base substitution). The C605R substitution removes a potential disulfide that was originally introduced to stabilize the SOSIP form of Env from viral strain BG505 [32, 37] involving cysteines that are maintained in the QH0692 gp140dsm form of Env [31, 33]. Since the adjacent residue 604 is also a cysteine, it is possible that the C605R substitution drives formation of an alternative disulfide involving C604.

Additive or synergistic effects on affinities for the 4E10 UCA were observed for combinations of amino acid changes. The W631R, C605R, and W623R mutations that all enhance binding individually, and that are the most abundant substitutions recovered from NGS sequencing of the sorted QH0692 library (Table 2), provide even higher affinities and numbers of sites when present as pairs. Combination of certain of these substitutions with additional mutations in the QH-17 and P6-8 clones isolated from the sorted library also resulted in enhanced 4E10 UCA binding (Table 3). Combination of the three most abundant substitutions (W631R, C605R, and W623R) with five additional mutations recovered in individual sorted variants (L545H, L566Q, C604S, A612T, and I642N) resulted in a form of Env that bound to the 4E10 UCA with higher affinity than any other tested variant (Fig 1F, Table 3).

Some substitutions that had no detectable effect on 4E10 UCA binding when present alone did affect binding when combined with other mutations. Despite having no effect as a single mutation, the substitution I642N enhances binding when present as a third mutation coupled to C605R and W631R (Table 3, Fig 1A and 1E). The three substitutions L545H, L566Q, and A612T that are present in the P6-8 variant (S1 Table) are not among the high frequency mutations in the NGS data and, thus, are unlikely to promote 4E10 UCA binding when present individually. However, they provide increased affinity for the 4E10 UCA compared to a variant containing only the C605R and W623R substitutions (Fig 1B and Table 2).

**Fig 3:**
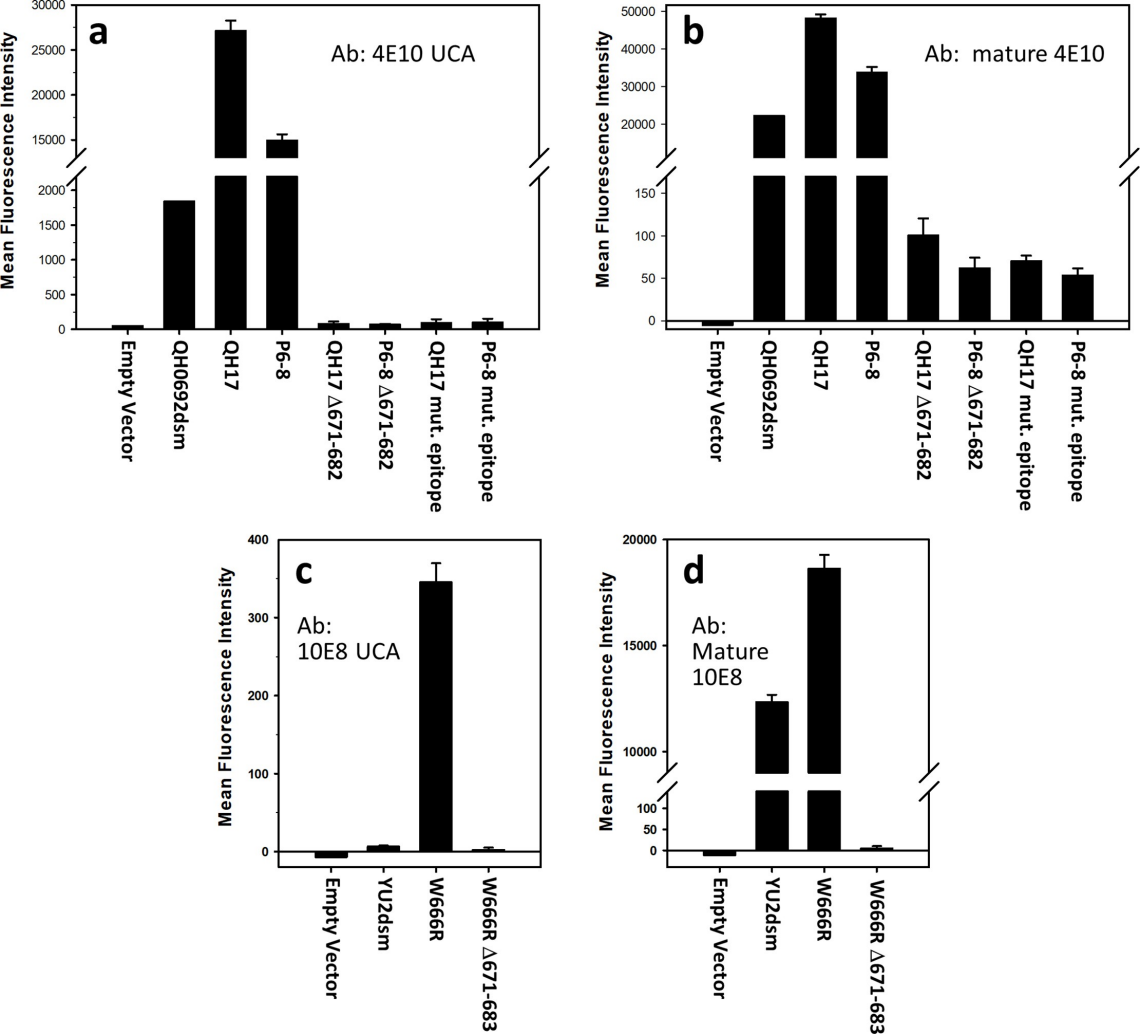
a) Effect of truncation (after W670) or mutagenesis (substitutions W672A F673A T675A) of the epitope for mature 4E10 on the binding of mature 4E10 to variant and otherwise unmutagenized forms of QH0692 gp140dsm; b) Effect of truncation (after W670) or mutagenesis (substitutions W672A F673A T675A) of the epitope for mature 4E10 on binding of the 4E10 UCA to variant and otherwise unmutagenized forms of QH0692 gp140dsm. c) Effect of truncation (after W670) of the epitope for mature 10E8 on binding of the 10E8 UCA to YU2 gp140dsm either containing or lacking the W666R substitution. d) Effect of truncation (after W670) of the epitope for mature 10E8 on binding of mature 10E8 to YU2 gp140dsm either containing or lacking the W666R substitution.

Mutations that enhance binding to the UCA form of 4E10 also improved binding to mature 4E10 (increasing both affinities and numbers of displayed sites) as well as to mature forms of four additional anti-MPER antibodies (Table 4 and S5 Fig). However, binding of antibodies to regions other than the MPER was either not affected or slightly diminished compared to the QH0692dsm reference form of Env. Binding of the 4E10 UCA to the identified Env variants was dependent on the presence of an intact form of the previously-identified epitope for mature 4E10 [38, 39], since removing this epitope by truncation of the gp140 construct at residue 671 or alteration of the epitope sequence by mutation resulted in loss of binding (Fig 3A). The presence of a large excess of synthetic peptide corresponding to the normal MPER region did not effectively compete the binding of the 4E10 UCA to the two tested Env variants (S6A and S6B Fig), consistent with the fact that the 4E10 UCA does not bind to normal forms of Env. Although removal of glycosylation sites enhances binding of some forms of Env to certain germline precursors of BNAbs [11, 26], a decrease in binding to the 4E10 UCA, was observed upon treatment of the variant containing the mutations C605R, W631R, and I642N with endoglycosidase H, which results in extensive deglycosylation of yeast-displayed Env [33], as indicated by loss of binding to the glycosylation-dependent antibody PGT126 (S7A Fig)

**Table 4:** Affinities of normal and 4E10 UCA-binding variant forms of Env for anti-HIV antibodies.

Strain	Mature VRC01 (anti-CD4bs)		Mature 447-52D (anti-V3 loop)		Mature 10E8 (anti-MPER)		Mature 2F5 (anti-MPER)		Mature Z13e1 (anti-MPER)		Mature 10E8 (anti-MPER)		Mature 4E10 (anti-MPER)		4E10 UCA	
	K_D_(nM)	Bmax^c^	K_D_(nM)	B_max_	K_D_(nM)	Bmax	K_D_(nM)	Bmax	K_D_(nM)	Bmax	K_D_(nM)	Bmax	K_D_(nM)	Bmax	K_D_(nM)	Bmax
**QH0692dsm**	42 ± 6	9800 ± 900	1.2 ± 0.1	38000 ± 400	240 ± 40	5600 ± 800	3.1 ± 0.4	28500 ± 1300	5.6 ± 0.3	22400 ± 400	240 ± 40	5600 ±800	8.2 ± 0.2	19700 ± 100	800 ± 60	7600 ± 500
**QH-17**^a^	34 ± 4	8300 ± 400	1.4 ± 0.1	38800 ± 800	60 ± 5	20400± 2000	0.8 ± 0.1	44400 ± 4000	1.2 ± 0.1	47900 ± 1800	60 ± 5	20400 ± 2000	1.6 ± 0.1	58000 ± 4400	44 ± 2	26900 ± 1100
**P6-8**^b^	47 ± 3	5400 ± 300	1.2 ± 0.01	27400 ± 600	37 ± 2	13800 ± 800	1.5 ± 0.1	37700± 1400	2.2 ± 0.3	43600 ± 2300	37 ± 2	13800 ± 800	2.2 ± 0.1	32000 ± 1400	61 ± 5	14100 ± 400

^a^The QH-17 variant contains the mutations C605R W631R I642N.

^b^The P6-8 variant contains the mutations L545H L566Q C605R A612T W623R.

^c^Reported B_max_ values are relative values based on saturation levels of fluorescence from fitting of saturation binding curves.

### Mutations in YU2 Env that confer binding to the 10E8 UCA

Following the second sort of the mutagenized library of YU2 Env variants, 27 out of 32 tested individual clones exhibited enhanced binding to the 10E8 UCA (S2 Table) but not to the control humanized DAC antibody (S11 Fig). Sequencing of individual clones and NGS sequencing of the sorted cells from a relaxed gate uncovered W666R as the predominant substitution, occurring in 24 of the 27 individual isolates and 28% of sequenced clones. In addition to W666R, 14 additional mutations were recovered at frequencies greater than three standard deviations above the mean frequency of mutations in the unsorted YU2-based library, though all were much less frequent than W666R. The spectrum of amino acid changes in the library sorted against the 10E8 UCA was quite different from that for 4E10; three of the most abundant 16 mutations were substitutions introducing arginine and five were substitutions introducing histidine. Aside from the most abundant W666R and W631R substitutions, none of the other most frequently recovered mutations were substitutions at tryptophan residues.

Clones from the sorted library that showed the highest level of 10E8 UCA binding in initial single point assays bind the 10E8 UCA with apparent dissociation constants of 70–90 nM, compared with undetectable levels of binding for the YU2dsm starting form of Env (Fig 2A). The W666R substitution alone was sufficient to confer binding of the YU2dsm Env to the 10E8 UCA with an apparent dissociation constant of ~120 nM (Fig 2B). In contrast to what was observed for mutations conferring binding to the 4E10 UCA, no lysine substitutions for W666 were identified among the NGS reads and site-directed substitution of lysine for the arginine at position 666 did not provide a significant enhancement in binding to the 10E8 UCA (Fig 2B).

Aside from W666R, no tested single amino acid substitution provided detectable Env binding to the 10E8 UCA, including the second most frequently recovered mutation, W631R, present among the NGS reads of the 10E8 UCA-sorted library at a frequency of 2%, which had provided the greatest enhancement of 4E10 UCA binding. Similarly, mutations at positions I682 and K683 that occur at some of the highest frequencies among the NGS reads (2.4% and 2.7%, respectively for all substitutions at each of these positions) and occur six times among the 27 characterized individual 10E8 UCA-binding clones show no detectable effect on 10E8 UCA binding when tested individually. Nonetheless, enhanced 10E8 UCA binding can be achieved in some Env variants that do not contain W666R, since clone C12 from the sorted library, which combines 9 different amino acid substitutions (none of which were present at high frequencies in the NGS of the sorted library; (S2 Table, Table 5) exhibits significant affinity for binding the 10E8 UCA (Fig 2C)

**Table 5:** Mutations recovered in NGS analysis of screen for binding to 10E8 UCA.

	Frequency of Recovery		
Amino acid substitution	Overall^a^	Presence of W666R^b^	Absence of W666R
W666R	0.2807	1.0000	0.0000
W631R	0.0240	0.0093	0.0297
E634D	0.0113	0.0147	0.0099
Q650H	0.0108	0.0163	0.0087
I682F	0.0107	0.0253	0.0050
Q577H	0.0104	0.0092	0.0109
Q658H	0.0094	0.0135	0.0078
K683M	0.0094	0.0158	0.0068
Q653H	0.0089	0.0092	0.0088
C605S	0.0088	0.0047	0.0105
E632D	0.0085	0.0154	0.0059
E560D	0.0085	0.0071	0.0090
C605R	0.0079	0.0067	0.0084
K683T	0.0079	0.0174	0.0041
S599Y	0.0076	0.0086	0.0073
N651H	0.0074	0.0088	0.0069

^a^from a total of 333,559 reads corresponding to sequences that appeared more than once in the NGS of the 10E8 UCA-sorted library. (The mutations shown occurred at frequencies more three standard deviations above the mean frequency (0.0023 ± 0.0017) for any particular nucleotide substitution in the corresponding unsorted QH0692 library. In cases where two nucleotide substitutions could provide the same amino acid change, the indicated double base substitutions occurred at frequencies greater than f_1_, where f_1_ = 3(√2)SD + 2M and M is the Mean and SD is the standard deviation of the unsorted library.

^b^from a total of 93,646 reads of sequences containing W666R that appeared more than once in the NGS of the 10E8 UCA-sorted library.

Several amino acid substitutions that have no effect as individual mutations nonetheless enhance 10E8 UCA binding when combined with W666R. In particular, W631R, I642N, and N656K (Table 6), all provide ~2-fold increases in binding in single point assays (p<0.01) compared to W666R alone and the combination of C605R, W631R, I642N, and W666R provides a higher level of binding than any of the paired mutations (Table 6). However, not all of the more frequently recovered substitutions in the NGS reads of the sorted library provide additive or synergistic effects with W666R (Table 6). It seems likely that some of the mutations recovered at frequencies of 2% or less among the NGS reads are enriched because they have synergistic or additive effects in combination with other substitutions that only moderately promote 10E8 UCA binding, as seen for combination of substitutions in clone C12.

**Table 6:** Effects of combinations of amino acid substitutions on 10E8 UCA binding.

Mutations^a^	Increase in 10E8 UCA binding when combined with W666R^b^
K500E	1.5 ± 0.3
K508N	1.3 ± 0.2
Q543L	1.3 ± 0.2
S546P	1.7 ± 0.3
L555M	1.1 ± 0.2
C605S	0.9 ± 0.2
C605R	1.1 ± 0.1
W614R	0.8 ± 0.03
D624V	1.5 ± 0.3
W631R	1.9 ± 0.2
E634D	1.1 ± 0.1
I642N	1.9 ± 0.1
N651Y	0.9 ± 0.2
N651H	1.5 ± 0.3
N656K	2.5 ± 0.4
W680R	0.9 ± 0.05
I682F	1.7 ± 0.3
K683T	1.2 ± 0.04
C605R W631R I642N	4.7 ± 0.3

^a^None of the listed mutations (other than W666R) exhibited any significant increase in 10E8 UCA binding when tested individually.

^b^Binding assayed in triplicate at a single concentration (340 nM).

The two best binding individual clones identified in the 10E8 UCA-sorted library exhibit, respectively, either no enhancement (clone C38) or a ~2-fold (clone C7) enhancement of the apparent affinity for binding to the mature 10E8 antibody compared to the starting YU2dsm form (S8 Fig, Table 7). However, these two clones do provide 5–10 fold enhancements of affinity for binding the mature anti-MPER antibodies Z13e1 and 4E10, compared to unmutagenized YU2dsm gp140. Neither clone exhibits detectable binding to the mature anti-MPER antibody 2F5, most likely because the W666R substitution present in both affects a critical residue in the previously-established 2F5 epitope (L^661^ELDKWASL^669^ [40–43]. Also, compared to un-mutagenized YU2dsm gp140, little change in the affinities of binding of 10E8 UCA-binding clones to two mature anti-gp120 BNAbs (VRC01 and 447-52D) was observed, but the clones exhibited weakened affinity for a third anti-gp120 BNAb, the mature form of the anti-V3 loop BNAb PGT126 (S8 Fig, Table 7).

**Table 7:** Affinities of normal and 10E8 UCA-binding variant forms of Env for anti-HIV antibodies.

Sample	Mature VRC01 (anti-CD4bs)		Mature 447-52D (anti-V3 loop)		Mature PGT126 (anti-V3 loop)		Mature 2F5 (anti-MPER)		Mature Z13e1 (anti-MPER)		Mature 10E8 (anti-MPER)		10E8 UCA		Mature 4E10 (anti-MPER)		4E10 UCA	
	K_D_ (nM)	B_max_^c^	K_D_ (nM)	B_max_	K_D_ (nM)	B_max_	K_D_ (nM)	B_max_	K_D_ (nM)	B_max_	K_D_ (nM)	B_max_	K_D_ (nM)	B	K_D_ (nM)	Bmax	K_D_ (nM)	B_max_
**YU2dsm**	50 ± 1	1420 ± 80	1.6 ± 0.03	11300 ± 300	80 ± 70	7900 ± 300	7.2 ± 0.3	15400 ± 200	9.4 ± 0.2	20500 ± 200	15 ± 0.1	18400 ± 200	ND	ND	5.1 ± 0.1	15400 ± 100	119 ± 5	7500 ± 200
**C38**^**a**^	47 ± 2	1360 ± 10	1.6 ± 0.1	18400 ± 500	900 ± 200	11700 ± 1300	ND	ND	1.8 ± 0.03	38700 ± 1100	4.3 ± 0.2	31700 ± 1000	70 ± 5	1700 ± 50	0.82 ± 0.02	35300 ± 1200	34 ± 1	15900 ± 700
**C7**^**b**^	96 ± 17	470 ± 20	1.9 ± 0.1	2600 ± 200	ND^d^	ND	ND	ND	1.1 ± 0.1	31100 ± 400	14 ± 1	18600± 600	90 ± 9	1400 ± 70	0.49 ± 0.01	31600 ± 1700	39 ± 2	18600 ± 500

^a^C38 contains the substitutions K500E K508N Q543L S546P D624V N651H N656K W666R I682F

^b^C7 contains the substitutions Q575K E584A S615T W666R K683T

^c^Reported Bmax values are relative values based on saturation levels of fluorescence from fitting of saturation binding curves.

^d^ND; Not Detected

As observed for variants binding the 4E10 UCA, truncation of the identified epitope [5] for mature 10E8 in the MPER region of YU2 Env blocked binding of variants to the 10E8 UCA (Fig 3C and 3D) but the presence of a large excess of MPER peptide did not block such binding (S6C and S6D Fig). Removal of N-glycosylation of the W666R mutation slightly diminished 10E8 UCA binding (S7B Fig).

The effects of the W666R substitution are not specific to the YU2dsm version of Env that was used for screening. The same substitution enhances binding of 10E8 UCA to a non-stabilized form of YU2, as well as to stabilized forms of Env from QH0692 and BG505 strains of HIV-1 (S9 Fig). Furthermore, the W666R mutation enhances 4E10 UCA binding both in both the QH0692 (Table 3) and YU2 (Table 7) backgrounds.

The W666R mutation results in introduction of a Lys-Arg sequence (only in the context of YU2 Env) with the potential to serve as a substrate for the yeast furin protease ortholog, Kex2p, which cleaves at dibasic residues. This raises the possibility that 10E8-UCA binding is the result of cleavage at this site. However, the context of the possible cleavage site is not optimal for Kex2p due to the presence of an Asp as the third residue to the N-terminal of the possible cleavage site [44, 45] and no additional cleavage at the introduced substitution was seen in immunoblots of Env variants with this mutation (S10 Fig). Furthermore, the 4E10 and 10E8 UCAs do not bind to the MPER peptide (see S8 Fig), so it would be surprising if cleavage that exposed the peptide would be sufficient to confer UCA antibody binding. The substitution W631R also introduces a Lys-Arg sequence in YU2 Env, however this mutation by itself does not result in increased 10E8 UCA binding (Table 6).

## Discussion

As a step toward developing immunogens capable of eliciting broadly neutralizing antibody responses to HIV, we used FACS-based sorting of a randomly mutagenized library of the variant forms of the HIV envelope glycoprotein to identify Env variants with increased affinities for UCA precursor forms of two anti-MPER BNAbs, 4E10 and 10E8. Sequence analysis of individual clones, as well as NGS sequencing of sorted populations, showed that a small number of amino acid substitutions dominated by certain Trp to Arg substitutions are responsible for most of the improvement in binding to both antibodies. Since most sites of mutations conferring UCA binding reside outside the regions defined as epitopes for mature forms of 4E10 or 10E8, the substituted amino acids must either contribute to formation of new epitopes recognized by the UCA antibodies or alter the configuration of the established epitopes of the corresponding mature antibodies. However, the epitopes of mature 4E10 and 10E8 must also contribute to the binding of the UCA antibodies, since removal or modification of the signature epitopes for the mature forms of 4E10 and 10E8 prevents binding of the identified variants to the UCA forms (Fig 3).

There are several indications that the observed increased binding of Env variants to UCA forms of BNAbs is due to the creation or exposure of new epitopes that are specific to each of the two UCA antibodies and is not simply the result of increased accessibility of epitopes on unfolded or degraded Env remnants: 1) Different mutations in Env have different effects on binding to the 4E10 UCA vs. the 10E8 UCA antibodies (and differential effects on the binding of mature anti-MPER antibodies), indicating that there is specificity to the interactions with each antibody. For example, neither of the two most frequently recovered mutations conferring enhanced binding to the 4E10 UCA (W631R and C605R; Table 2) provided any enhancement of binding to the 10E8 UCA when present as single mutations. 2) The increased binding is due to substantial increases in apparent binding affinity and not just increases in the numbers of sites that could reflect binding to partially denatured forms of Env. 3) The UCA forms of 4E10 and 10E8 do not bind to an MPER peptide containing the established epitopes for the mature forms of the antibodies, since binding of the UCA antibodies to Env variants is not competed by high levels of the MPER peptide. Thus, simple exposure of the MPER region in denatured Env would not be expected to be sufficient to confer binding. Nonetheless, the established epitopes for mature forms of 4E10 and 10E8 must contribute to variant binding to the UCA antibodies, since alteration of those epitopes diminishes binding (Fig 3).

Despite the evidence for specificity of their effects, the partial overlap between the sets of mutations conferring variant binding to the 4E10 and 10E8 UCAs, as well as their effects on binding to mature anti-MPER antibodies, suggests that the mutations may also alter the accessibility of the gp41 moiety of Env for antibody binding. When present as a single mutation, W666R increased binding to both the 4E10 and 10E8 UCAs. Furthermore, several of the mutations that most strongly enhance 4E10 UCA binding act synergistically with additional mutations to enhance binding of the 10E8 UCA, although they do not enhance 10E8 UCA binding as single mutations. Increased accessibility of epitopes in the variant forms of Env is also suggested by the observed increases in the apparent number of binding sites for UCA and mature forms of anti-MPER antibodies on yeast cells. These increases are not due to overall increases in expression or trafficking, since anti-V5 epitope antibody binding to the variant forms of Env is not significantly altered by the mutations (S4 Fig). However, some of these apparent increases could result from reduced dissociation of the higher affinity antibodies from the yeast cells during the non-equilibrium binding assay.

It is not currently clear how mature anti-MPER antibodies gain access to a region of Env that, in the context of a stable trimer, is expected to be largely sequestered at the membrane surface. The MPER region of gp41 appears to be flexible, based on the diversity of conformations observed in different crystal structures [46–48]. During viral fusion with cell membranes, gp41 is thought to undergo a conformational transition to a pair of helices that forms a fusogenic six helix bundle in trimers [49, 50]. Antibody binding may occur through a conformational change to a “fusion-intermediate” or “pre-hairpin” state [47, 48, 51, 52] on the pathway to the fusogenic conformation. Extraction of target sequences from the membrane may also be promoted by binding to anti-MPER antibodies [53] and by displacement of the gp120 moiety away from the membrane surface [47].

**Fig 4:**
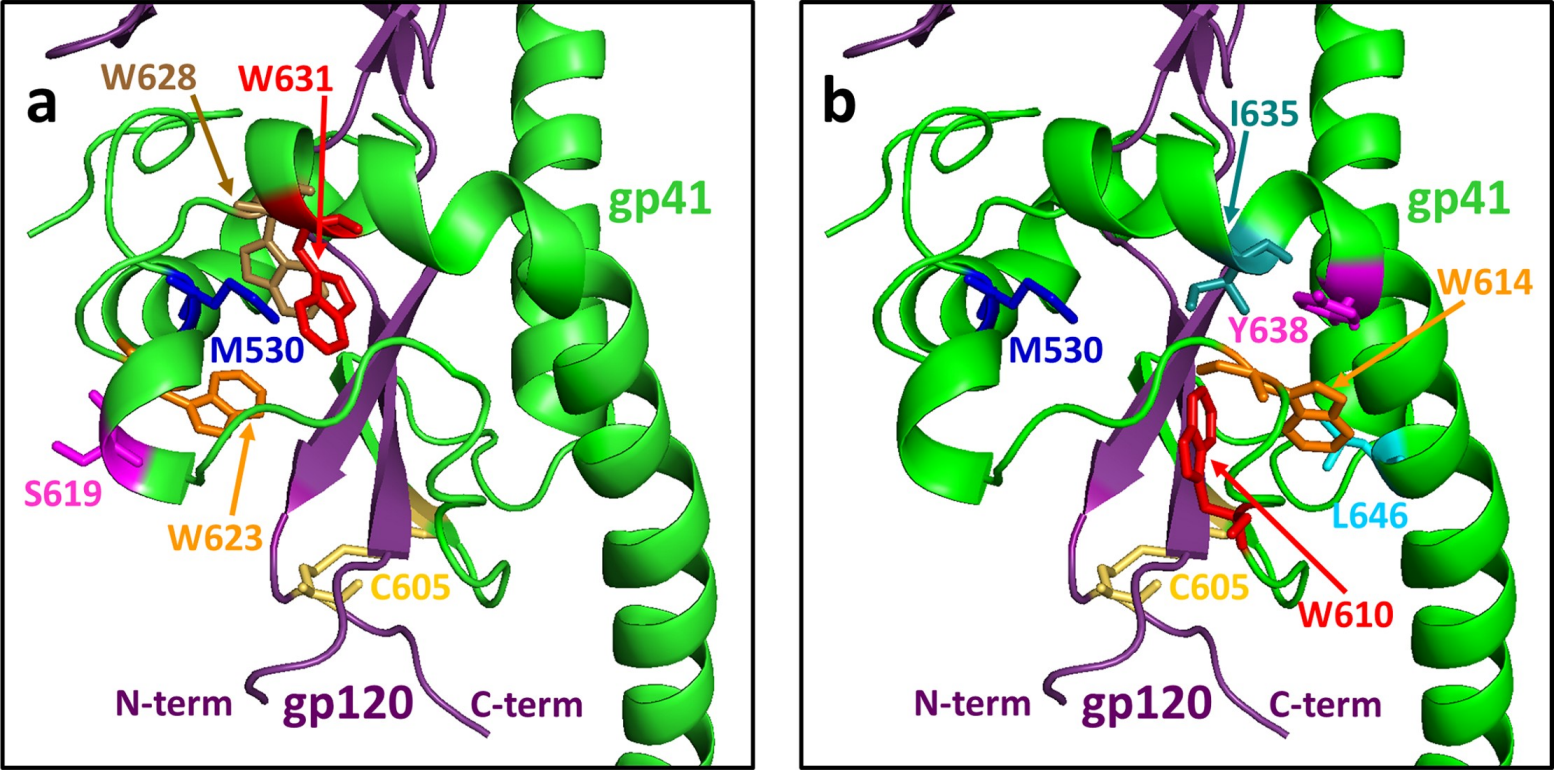
a) Nine of the ten most frequently identified mutations conferring Env binding to the 4E10 UCA map to the “clasp” region of Env that is proposed to lock the protein in the pre-fusion state. a) Sites corresponding to mutations Q619R, W623R, W631R, and W628R, four of the most abundant mutations from the NGS sequencing of the sorted library (see Table 2) are located directly in the M530-tryptophan clasp. The mutated residues are indicated in color-coded stick representations corresponding to the residues indicated on the figure. Residues M530 and C605 (as a disulfide with C501) are also indicated for reference. The displayed structure is the presumed pre-fusion form of the BG505 SOSIP.664 Env Trimer (PDB code: 4TVP [56]) in complex with antibodies PGT122 and 35O22 (not shown in this figure). Note that position 619 is a serine in Env from strain BG505, as indicated, but is glutamine in Env of strain QH0692. Gp41 is shown in green, gp120 in dark purple. b) Sites corresponding to mutations C605R, W610R, W614R, Y638H, L646S, map to the interface between gp41 and the N- and C-terminal loop of gp120 near the M530 clasp (same view of Env as panel a, with different residues highlighted).

**Fig 5:**
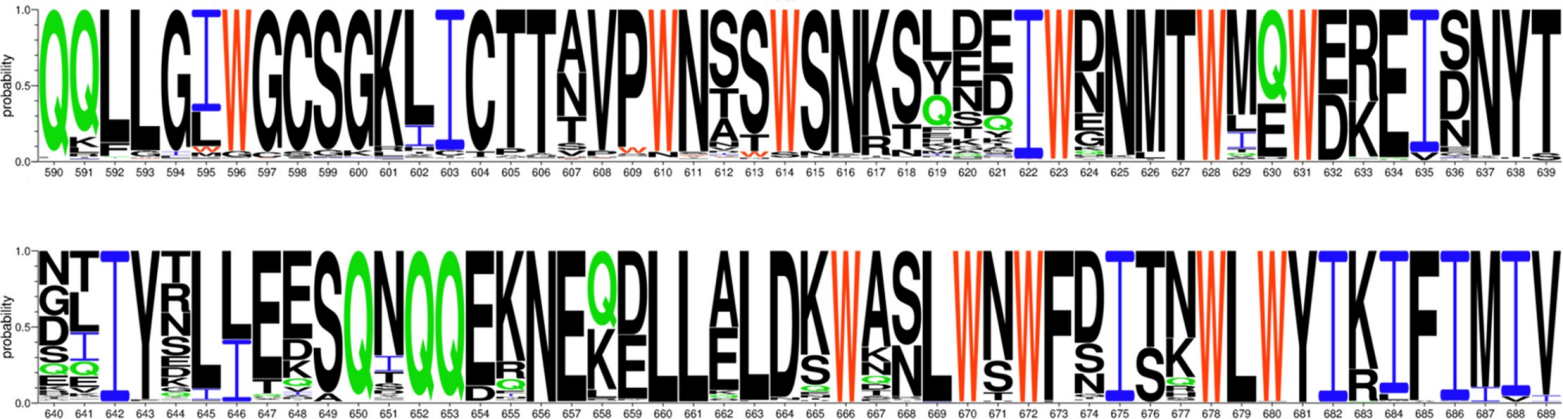
Trp-rich sequence motifs in the gp41 region of Env. The figure was generated using the AnalyzeAlign database of the Los Alamos National Laboratory (http://www.hiv.lanl.gov/) based on the curated alignment of 6636 sequences. Trp residues are shown in red, Gln residues in green and Ile residues in blue. Abundances of selected residues are shown in S3 Table.

Available three dimensional structures of mature 4E10 and 10E8 antibodies in complex with peptides corresponding to the MPER region and with a scaffolded version of the 10E8 epitope do not include the particular sites in gp41 at which we have identified relevant mutations [54, 55] and only a low resolution structure of mature 10E8 in complex with a native Env trimer complex is available [47]. Thus, we mapped the sites of amino acid substitutions promoting binding UCA antibodies onto available structures of gp41 solved in the absence of anti-MPER antibodies. Substitutions that promote binding to the 4E10 UCA map to a region of gp41 that appears to stabilize the pre-fusion state through the formation of a “clasp” formed by hydrophobic interactions of M530 with a “tryptophan sandwich” consisting of residues W623, W628, and W631 [46, 56]. These three tryptophans are located on a four-helix bundle of gp41 that wraps around an inserted pair of strands containing the N- and C-terminal regions of gp120. The mutations, Q619R, W623R, W628R, and W631R, which were among the most frequently identified substitutions in the library sorted for 4E10 UCA binding, map directly to the tryptophan cluster (see Fig 4A). Other commonly recovered mutations from this library, C605R, W610R, W614R, Y638H, and L646S map to the nearby interface between gp41 and the inserted N- and C-termini of gp120 (Fig 4B). Together, these nine substitutions comprise all of the most frequently recovered substitutions identified in the 4E10 UCA-sorted library, aside from the mutation W666R, which is in a region of gp41 that is not present in the pre-fusion state structures (see below). The tryptophan residues in this region as well as the isoleucines at positions 635 and 642 are tightly conserved across HIV strains and the isoleucine at position 646 is conserved as isoleucine or leucine (Fig 5). Based on these structural considerations, each of these mutations, particularly those that substitute charged arginine residues for a tryptophan, is likely to destabilize the pre-fusion conformation, releasing the clasp, and allowing for greater exposure of gp41 for antibody binding. In fact, alanine substitutions for each of the clasp-region residues W610, W614, W623, W628, W631, I635, and I642 reduce gp120/gp41 association, reduce the thermodynamic stability of model helical gp41 peptides, and/or reduce the ability of Env to promote cell fusion in a mammalian expression system [57–59].

**Fig 6:**
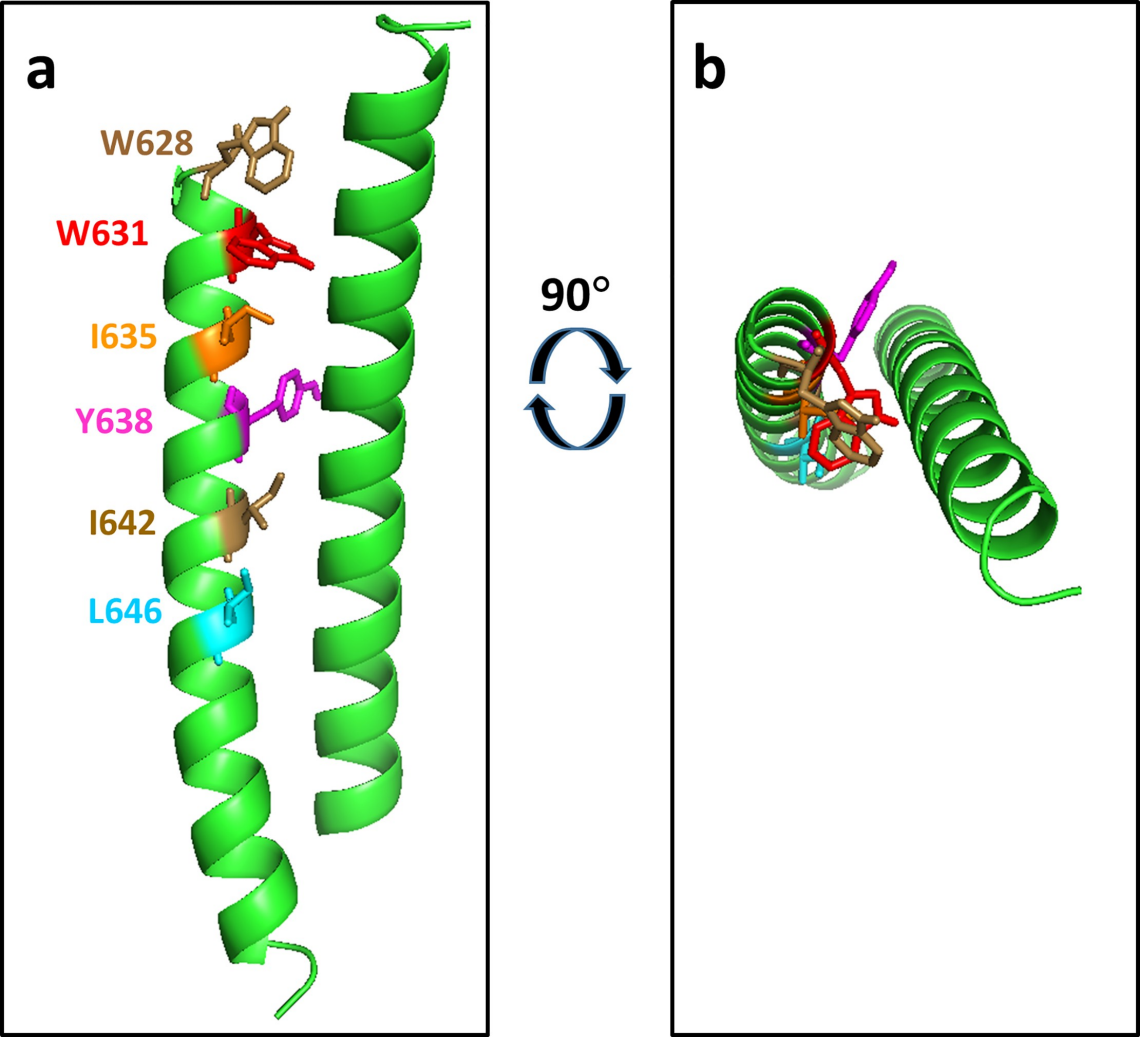
Frequently identified mutations conferring Env binding to the 4E10 UCA map to the hydrophobic groove between adjacent helices in the six-helix, fusion-active form of gp41. a) Mutated residues indicated on the structure of protease cleaved gp41, which consists of a complex of peptides corresponding to residues 546–581 and 528–661 of Env (HXB2 numbering). Two helices from the overall six helix structure (PDB code: 1AIK [49]) are shown. This structure is reported to be very similar to other structures of putative fusion-active forms of gp41 [56]. The sites of the W628R, W631R, I635, Y638H, and L646S, frequently-recovered mutations among NGS reads are indicated as color-coded stick representations. Note that other frequently-recovered mutations are at sites outside the region of sequence encompassed by this structure. One additional indicated site, I642, was recovered less frequently, but was shown to modulate binding to the 4E10 UCA when mutated together with more frequently recovered substitutions (see text). b) 90° rotated view of the structure shown in a).

Some of the mutations that confer binding to the 4E10 UCA also map to critical regions of the structure of the fusion-active conformation of gp41 (Fig 6), which consists of a trimer of paired helices in a hairpin configuration [49, 56]. The most frequently-recovered substitutions from the 4E10 UCA library, W628R, W631R, Y638H, and L646S localize to one helical face that forms a hydrophobic groove mediating assembly of the six helix structure [58]. I635 and I642, positions at which mutations act together with W631R to promote 4E10 UCA binding, also map to this same helical face. (The other most frequently recovered mutations in the sorted library are located at sequence positions that are not included in the available fusion-active structures.) The importance of these residues in membrane fusion is underscored by previous observations that alanine substitutions at many of these positions block fusion in transfected cell systems [58, 59].

**Fig 7:**
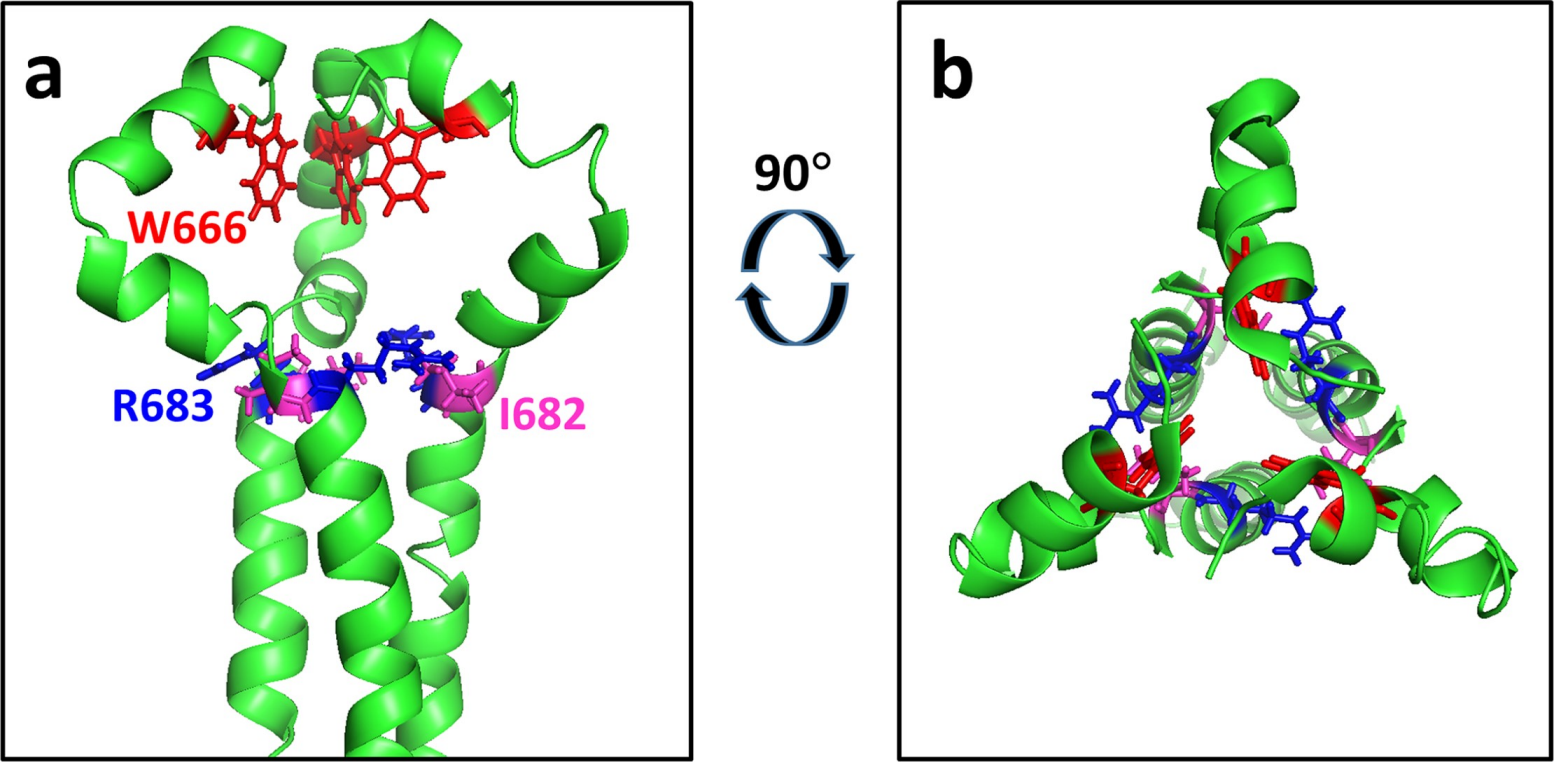
W666R, the most frequently recovered mutation conferring binding to the 10E8 UCA maps to the trimer interface of the putative pre-fusion structure of the MPER region and the adjacent transmembrane domain. The figure is based on the NMR structure of residues 660–710 (HXB2 numbering) of Env from HIV strain 92UG024.2 reconstituted into dimyristoylphophatidylcholine/dihexanoylphosphatiylcholine (DMPC/DHPC) bicelles. (PDB code 6E8W [48]). a) The position of W666R is shown as a stick representation in red. The two positions (I682 and R683) of three mutations that were frequently recovered in the 10E8 UCA-sorted library are also indicated. These are the only three of the most abundant mutations (Table 5) that map to the sequences included in this structure. Note that position 683 is occupied by lysine in the envelope from the YU2 strain but is arginine in Env from strain 92UG024.2. a) Representation viewed parallel to the membrane surface. b) Representation viewed perpendicular to the membrane.

W666R, the primary mutation conferring 10E8 UCA binding that also promotes binding to the 4E10 UCA, is a replacement of one of five strongly conserved tryptophan residues in the C-terminal portion of gp41 (see Fig 5 and S3 Table) [60]. An NMR structure in lipid bicelles of the MPER region in combination with the predicted transmembrane segment shows W666 providing critical contacts in a trimeric assembly that is proposed to constitute a pre-fusion conformation (Fig 7)[48]. (Available structures of the intact Env ectodomain do not include W666, as they all terminate at or before residue 664.) In a crystal structure of gp41 alone, in what is proposed to be a late fusion state, W666 was observed to contribute to solvent-exposed hydrophobic surface involved in inter-helical interactions of the six-helix fusion structure [61]. I682 and K683, two other sites at which mutations were found at high frequencies in the 10E8 UCA-sorted library, also map to close contacts among the constituent subunits of this trimer (Fig 7) (though single substitutions at these sites do not enhance 10E8 UCA). The functional importance of these sites is supported by demonstrations that the substitutions W666A, W666R, and K683A all severely diminish viral infectivity [61, 62].

The positions of the mutations that we have identified with respect to the available structures, along with previous mutagenic studies of the affected residues, indicate that the non-conservative mutations that confer UCA anibody binding destabilize the normal pre-fusion conformation of gp41. Such structural changes in Env, particularly those induced by engagement of the CD4 receptor, have previously been implicated in enhancing exposure of epitopes for anti-MPER antibodies that are normally obscured by gp120 and the viral membrane [48, 53, 63, 64]. Destabilization of the pre-fusion state might be expected to favor the fusion-active alternative structure, however mapping of the mutation sites to the putative six-helix fusion-active structure indicates that these same mutations could also destabilize that structure. This could allow the mutated regions of gp41 to adopt diverse alternative conformations, including some with enhanced exposure at the surface of Env, that provide enhanced recognition by the 4E10 and 10E8 UCAs. Such transitions to alternative conformations of gp41 may also be promoted by mutations that we identified that have no effect when tested as single substitutions but exhibit additive or synergistic effects when present in Env variants containing additional substitutions that promote anti-MPER UCA binding.

The specificity and breadth of the mutagenesis and screening procedures that we used in this study allowed recovery of thousands of independently-derived variants, each containing different combinations of mutations that enhance UCA binding, apparently by simultaneously destabilizing both the pre-fusion state and the fusion-active hairpin states. The breadth of effects of the identified mutations raises the possibility that approaches like the one described here could be used to develop optimized immunogenic forms of Env that enhance binding to multiple UCAs and mature antibodies. The diversity of the recovered mutants also reinforces proposals to use similar random mutagenic approaches for rapid analysis of amino acids required for specific antibody binding [65]. NGS analysis of the library sorted against the 4E10 UCA uncovered more than 100 mutations that appeared more than 10-fold less often in clones containing the W631R mutation than in clones lacking W631R. The relative depletion of these substitutions from W631R-containing variants appears to result from their incompatibility with 4E10 GEP binding, either because they inhibit formation of the new 4E10 UCA-binding epitope or because they cause misfolding of Env. For example, the non-conservative substitution I635K is found at a frequency of only 0.02% among NGS reads from the 4E10 UCA-sorted library that also contain the additional substitution W631R, despite being present at the relatively high frequency of 2% among reads that do not contain the mutation W631R. Additional non-conservative substitutions at this position, I635N, I635M, and I635T, are found, respectively 328-, 68-, and 13-fold less frequently in reads containing W631R than in reads lacking W631R. However, the conservative substitution I635V is found at approximately equal frequencies in reads lacking or containing W631R and the substitution I635L is found 91-fold more frequently in reads containing W631R. Thus, in Env variants containing W631R, non-conservative substitutions for I635 apparently diminish the positive effects of W631R on binding, while conservative substitutions at this position can actually promote 4E10 UCA binding. (Note that comparing mutation frequencies within a single library between the variants that contain or do not contain the W631R substitution removes biases from factors related to the frequency of codon-specific amino acid changes.)

The immediate goal of this study was to identify candidate immunogens that would be useful for priming the activation of B cell precursors expressing UCAs of BNAbs. It has been suggested that priming with such a precursor-binding immunogen, would need to be followed by subsequent boosts with more mature forms of Env to drive the somatic hypermutation necessary to achieve maturation to a potent and broad antibody response capable of effectively neutralizing circulating forms of Env [4, 29]. However, a notable result of our studies is that the Env variants that we identified showing enhanced binding to UCA forms of 4E10 and 10E8 generally also exhibit enhanced binding to mature forms of these antibodies, raising the possibility that these variants could be used both for priming and boosting.

While there are often discrepancies between the immunogenicity of a particular candidate vaccine and its antigenicity (or affinity) toward the antibody it is designed to elicit, the approach described here addresses differential binding to precursor vs. mature antibodies as a potential major source of such discrepancies. Full characterization of the immunogenic potential of the UCA-binding Env variants that we have identified will require future *in vivo* testing of antibody responses. To achieve the desired anti-MPER immune responses, it may eventually be necessary to take steps [66–69] to moderate possible effects of the reported autoreactivity of anti-MPER antibodies [68, 70–72], which could lead to inhibition of the maturation of B cells with the desired anti-MPER specificities.

It will be also be important to determine whether the binding properties of the identified variants are particular to the yeast display system, or whether they can be recapitulated using the same variants expressed in mammalian cells, where glycosylation patterns may be somewhat different from yeast. However, to be effective in activating an initial immune response, forms of Env used as immunogens do not need to be identical to the authentic forms found in viral envelopes. It may, in fact, even be possible to stimulate the desired immune responses with whole antigen-displaying yeast cells [73–76]. Since there is likely to be evolutionary pressure on viruses such as HIV to evolve envelope proteins that fail to bind to the precursors of BNAbs, screening in a heterologous system such as yeast provides a way of identifying variants with useful immunogenic properties due to mutations that would render Env nonfunctional in the context of an intact virus. Consistent with this, we find that mutations that confer Env binding to the UCA forms of the anti-MPER BNAbs involve substitutions at amino acid positions that are highly conserved among different HIV isolates and appear to be involved in stabilizing functionally important conformations of Env.

## Methods

### Antibody production

The following anti-HIV envelope antibodies and additional reagents were obtained from the NIH AIDS Reagents Program Division of AIDS, NIAID, NIH (original providers indicated in parentheses): 10E8 (Dr. Mark Connors)[5], 4E10 (Polymun Scientific)[39], MPER peptide (Division of AIDS, NIAID, NIH), PGT126, (International AIDS Vaccine Initiative)[14], Z13e1 (Dr. Michael Zwick)[30]. Antibody 447-52D was expressed from a synthetic gene in HEK293 cells and purified essentially as described [77]. Mouse anti-V5 epitope antibody was from Pierce (ThermoFisher) and the goat anti-gp120 polyclonal antibody was from Meridian Bioscience. Plasmids encoding the gHV and gLV reconstructed UCA form of antibody 10E8 with a mature CDRH3 region [78] were obtained, from Dr. Peter Kwong of NIAID. The UCA form of 4E10 was reconstructed as described previously [33]. Cloning, transfection of HEK293T cells, and purification of reconstructed precursor antibodies were performed as described previously [77]. The anti-interleukin 2 receptor (CD25) antibody daclizumab (DAC) [36]was used as a non-Env-binding negative control for detection of possible enhancement of non-specific antibody binding.

### Yeast surface display and binding assays

Plasmids encoding the stabilized gp140 form of HIV strains YU2, QH0692 and BG505 and the non-stabilized form of YU2 for yeast surface display were constructed and cloned into plasmid pYD5 [79] as described previously [33].

The induction protocol for expressing HIV envelope proteins at the cell surface of yeast strain EBY100 [34] was as described previously [33]. In summary, cells of the appropriate strains were first cultured in unbuffered synthetic complete medium lacking tryptophan with raffinose as the sole carbon source, then regrown at 30˚C in the same media buffered to pH 6 with 0.1 M sodium phosphate to an OD_600_ of 0.1–0.2, followed by addition of galactose (2% final concentration) to induce Env expression. After culturing for an additional 23–25 hours at 20˚C, the cells were pelleted, washed with PBI (phosphate buffered saline (pH 7.2) containing 1% BSA and protease inhibitors (EDTA-free protease inhibitor tablets by Roche or Pierce, prepared as directed by the manufacturer)), and used for binding assays either immediately or after brief storage at 4˚C.

Antibody binding to cells expressing envelope protein was measured using flow cytometry, essentially as described [33]. Briefly, 0.5–1 × 10^6^ cells were incubated overnight at 4˚C with anti-Env or anti-V5 epitope antibodies in PBI. The primary antibody concentrations used are indicated in the relevant figure legends. Cells were then washed with ice-cold PBS containing 1% BSA and then incubated with 25–50 nM Dylight550-, Alexa633- or Alexa647-conjugated secondary antibodies in PBI at 4˚C or room temperature for 1 hour. Cells were pelleted, resuspended in 0.3–0.4 ml ice-cold PBS and kept on ice or at room temperature while conducting flow cytometry with a 12- or 18-color LSRII (Becton Dickinson) using either a 532 nm laser with detection at 563–587 nm or a 633 nm laser and a detection channel of 640–680 nm. Competition of antibody binding by 23-mer MPER peptide was conducted by mixing the peptide with the cells prior to incubation with the primary antibody.

Results from flow cytometry were analyzed using the program FlowJo. Cells were gated for singlets using forward and side scattering and mean fluorescence intensities were calculated from scatter-gated cells. Saturation and competitive binding data were further analyzed using the nonlinear least squares functions of Sigmaplot assuming single site binding. Apparent dissociation constants, relative B_max_ values, and associated standard errors for saturation binding data were determined by averaging separately-fit dissociation constants and B_max_ values from 3 independent yeast transformants. The fit parameters are presented as apparent K_D_ and B_max_ values, since they are not measured under true equilibrium conditions and, for example, rapid off-rates for binding could diminish the apparent dissociation constants and B_max_ values. Relative B_max_ from binding data values refer to arbitrary levels of fluorescence and are presented for purposes of comparison only. Statistical evaluation of fluorescence values against control samples was conducted by one-way ANOVA using Dunnett’s post-test. None of the tested forms of Env showed any detectable binding to the DAC anti-human isotype control antibody (S12 Fig).

### Mutagenesis, library construction, and library screening

The mutagenized QH0692dsm library was created by error-prone PCR of the gp41 region of HIV Envelope using the Genemorph II Random Mutagenesis kit (Agilent) with 0.4 or 1.5 μg template. The two oligonucleotides used for amplification annealed approximately 100 nucleotides upstream and downstream of the region targeted for mutagenesis (the upstream primer (ON1835) sequence corresponds to amino acids 474 to 484 of the envelope protein from HIV strain QH0692 and the downstream primer (ON1836) sequence is complementary to the nucleotides coding amino acids 1 to 10 at the beginning of the *AGA2* sequence, approximately 100 bp downstream of the EcoRI site of plasmid pYD5. The resulting PCR product was then re-amplified using the same primers and Taq polymerase to obtain greater quantities, keeping the template concentration sufficiently high (100ng/200μl) to maintain the library diversity. A pYD5 vector containing the gene for QH0692dsm gp120 fused to the V5 epitope and *AGA2* (pMD2671) was linearized with EcoRI, which cuts at the 3’ end of gp120. The PCR product (12 μg total from the amplified 0.4 μg and the 1.5 μg template-containing mutagenesis reactions at a 1:1 ratio) along with 4 μg of the EcoRI-linearized pMD2671 (that has ends that are homologous to the ends of the PCR product) were then used to transform ~7 × 10^8^ EBY100 cells by electroporation (Bio-Rad GenePulser at 1 kV, 25μF in a 0.2 cm gap cuvette), essentially as described previously [80] such that intact plasmids were formed through homologous recombination (S2 Fig). After transformation, library size was determined by plating a small portion of the transformed cells, after a 1 hour rescue incubation at 30°C, and was determined to be 2.3 × 10^7^. The remaining electroporated cells were pelleted, resuspended in 100 ml SD-Trp media, and allowed to grow overnight to an OD_600_ of ~2. The library was subjected to one more round of growth to further reduce the presence of any untransfomed cells by innoculating 200 ml SD-Trp with 10 ml of the overnight culture and growing to an OD_600_ of ~2. The library was then stored in 7% (v/v) DMSO at -70°C, in aliquots. For further use, aliquots were thawed at room temperature just prior to being cultured.

The unmutated form of YU2 dSM gp41 library insert was generated by PCR with Phusion DNA polymerase (NEB) following supplier’s protocol using plasmid encoding YU2dsm gp140 as template. The region amplified by PCR spanned from amino acid 474 (HXB2 numbering) of the envelope protein to a linker in the plasmid vector 31 amino acids away from the C-terminal of YU2dsm gp140), encompassing 760 bp. PCR products were gel-purified and 40 ng were used as template for error-prone PCR to prepare mutagenized insert. Error-prone PCR protocol was performed using Agilent Genemorph II random mutagenesis kit as described previously [81] with modifications. Briefly, 84 ng of gel-purified insert was incubated with 7 mM MgCl_2_, 5 mM MnCl_2_, 1 mM dCTP, dTTP and 200 μM dGTP and dATP, 2.5 units of Mutazyme II and 20 pmole of oligonucleotide primers in a volume of 50 μl. The mutagenized product was gel-purified and 7 μg were co-transformed into yeast with 1 μg of vector that had been digested with *Eco*RI, as described above. The library size was determined to be ~10^7^. Prior to screening, plasmids were rescued from randomly selected colonies of the library and sequenced to determine the mutation rate. The rest of the transformed cells were grown in 100 ml SD-Trp medium for approximately 24 hours before freezing.

The high mutation rates of the mutagenized libraries (0.62% and 0.65% for the QH0692 and YU2 libraries, respectively) led to an increased proportion of cells that fail to display the V5 epitope, compared to un-mutagenized cultures (S2 Fig). This increase is due to the mutagenic introduction of stop codons and mutations that affect the folding or secretion of Env. (A background of cells that fail to display the V5 epitope resulting from spontaneous plasmid loss is present in both mutagenized and un-mutagenized cultures.) However, the reduction in V5-expressing clones was less than 50% for both screened libraries. The listed mutation rates for the libraries include a contribution from sequencing errors of 0.2%, estimated based NGS sequencing analysis of unmutagenized YU2dsm Env. The NGS confirmed that the average mutation rates were uniform over the mutagenized regions, though they varied ~2-3-fold between adjacent positions (S11 Fig).

Libraries of HIV envelope proteins were induced for expression and prepared for antibody binding, as described above. A population of cells at least 10-fold greater than the size of the library was incubated for 14–18 hours at 4°C with the human UCA form of 4E10 at a concentration of 250 nM or with UCA form of 10E8 at a concentration of 340 nM, and simultaneously with mouse antibody against the V5 epitope, followed by sequential incubations with Alexa633 or Alex647-labeled anti-human and Alexa546-labeled anti-mouse antibodies at room temperature or 4 ˚C for 1 hour. Cells were washed once before resuspending in PBS to 3 × 10^7^ cells/ml for sorting by a FACSAria II (BD Biosciences) using a 532 nm laser with detection at 550–600 nm and a 640 nm laser and a detection channel of 656–684 nm at the same time. Cells were gated by forward and side scatter, and 1% to 5% sorting gates were used in sort cycles. Typical sorting gates for initial and later selection rounds are shown in S3 Fig. Sorted cells were captured in SD-Trp medium and grown for 12 to 24 h for subsequent sorting rounds or analyses of selected pools.

Site directed mutagenesis was performed as described [82, 83].

### Plasmid rescue and sequencing

Clones recovered from library sorts were cultured in unbuffered synthetic complete medium lacking tryptophan (SD-Trp) for overnight growth at 30˚C. Plasmids were then isolated using a modification of the manufacturer’s protocol for the “Wizard® Plus SV Minipreps DNA Purification System” (Promega), as described [84]. Upon sequencing of the sorted clones from QH0692dsm library, it was discovered that the starting plasmid for mutagenesis had inadvertently contained a mixture of two alleles, one containing the substitution G459V, and the other lacking this substitution. However, reconstructed forms of Env without G459V bound well to the 4E10 UCA and variants used for further studies did not contain this mutation.

To recover plasmids from library pools for NGS sequencing, QH0692dsm, YU2dsm libraries and sorted pools were cultured in SD-Trp for overnight growth at 30 ˚C. After pelleting cells, yeast plasmids were rescued using the Zymoprep Yeast Plasmid Miniprep II kit (Zymo Research) following supplier’s protocol. For QH0692dsm libraries, NGS sequencing primers contained Illumina specific adapters and indices aligned to HXB2 amino acid position 540 of the envelope protein and V5 tag in the vector, respectively, spanning 448 bp in total. For YU2dsm libraries, sequencing primers were aligned to HXB2 position 520 of the envelope protein and V5 tag in the vector, respectively, spanning 504bp in total. Sequencing samples were prepared by PCR using plasmids rescued from libraries as template, primed with next-generation sequencing primers, with Phusion DNA polymerase (NEB) following supplier’s protocol. Samples were submitted to the University of Rochester Genomics Research Center where Qubit Fluorometric quantitation (ThermoFisher) and Bioanalyzer (Agilent Technologies, Santa Clara, CA) sizing, quantitation and quality control was performed before being transferred to the flow cell and sequenced using the MiSeq system (Illumina, Inc., San Diego, CA).

### Analysis of sequences

Sequencing reads were analyzed using a custom Python script. Read lengths for the QH0692 and YU2 libraries were 448 and 504 nucleotides, respectively. The total numbers of reads for the unsorted QH0692 and YU2 libraries were 679,954 and 433,247, respectively. After filtering out sequences with gaps and frameshifts, the numbers of reads were reduced to 588,301 for QH0692 and 395,860 for YU2. The average rate of substitution for the unsorted QH0692 library was 0.62% per nucleotide, yielding an average of 3600 reads with substitutions at each position. The corresponding average substitution frequency for the unsorted YU2 library was 0.65%, yielding an average of 2600 reads with substitutions at each position. The sequencing error rate was ~0.2%, based on the apparent mutation rate of an NGS analysis of un-mutagenized YU2dsm. The apparent mutation rates of different sequenced pools were not corrected for this number.

The total numbers of reads for the sorted QH0692 and YU2 libraries were 885,276 and 636,761, respectively, which, after filtering for intact reading frames, were reduced to 560,076 and 333,559. Since the number of sequencing reads in the sorted libraries greatly exceeded the number of cells in the sorted population, any read that is only present once likely represents a sequencing error. Thus, to minimize effects of sequencing errors on analysis of sorted libraries, reads from these libraries were filtered to remove sequences that only appeared once, leaving 333,559 reads encoding 16,842 different amino acid sequences that were each recovered more than once for the sorted QH0692 library and 560,076 reads encoding 20,288 different amino acid sequences for the sorted YU2 library. To estimate the significance of enrichment of substitutions in the post-sort libraries, frequencies of recovering particular substitutions were compared with a 3-sigma threshold (average plus three standard deviations) based on the mean frequency and standard deviation of that particular substitution in the corresponding unsorted mutational library.

### Glycosidase treatment

Glycosidase treatment of yeast displayed envelope proteins was conducted by incubating 6–8 × 10^5^ galactose-induced cells with EndoH (New England Biolabs), at a concentration of 20 U/μl, in the manufacturer-recommended buffer (50 mM Sodium Citrate (pH 5.5)) at 37˚C for 2–5 hours. The cells were then pelleted and washed with PBS containing 1% BSA before incubation with antibodies and flow cytometry, as described above.

### Immunoblotting

For immunoblotting, cells were pelleted and resuspended in loading buffer containing 100mM DTT (prepared as described [33]) at 1x10^8^ cells/ml. Env protein was released from the surface of the cells by the DTT-mediated reduction of the disulfide bonds between Aga1p and Aga2p. The protein released from approximately 7 × 10^5^ cells was loaded per lane on a Bio Rad Criterion 4–15% gradient gel. (The sample from empty vector containing strain A4793 was diluted an additional 100 fold before loading.) Bands were transferred to a 0.2 μm nitrocellulose membrane and blocked in PBS containing 5% non-fat milk for 2 hours. The membrane was then incubated with 0.1 μg/ml anti-V5 epitope antibody (BioRad) in PBS containing 3% BSA and 0.1% Tween-20 for 2 hours, washed 3 times for 10 minutes in PBS, incubated for 2 hours with anti-mouse HRP-conjugated secondary antibodies (Biolegend) at a 1:6000 dilution in PBS containing 5% non-fat milk and 0.1% Tween-20, then washed three times in PBS before visualizing by enhanced chemiluminescence using Supersignal West Dura Extended Duration Substrate, following the manufacturer-recommended protocol. All incubations were performed at room temperature.

### Structural representations

Structural figures were generated using PyMOL Molecular Graphics System, Version 2.0 Schrödinger, LLC, based on the referenced PDB entries.

## Supporting information

S1 Fig Alignment of Env amino acid sequences used in this work. N-terminal signal sequences have been removed, since these were replaced by the signal sequence from Aga2p. The hydrophilic fusion peptide sequence (highlighted in green) and the optimized Kex2p-cleavage site (highlighted in purple) are as described [31]. Additional “stabilizing” mutations as described by Grimm et al., are highlighted in cyan. “SOSIP” mutations [85] are highlighted in grey and the original BG505 SOSIP sequence is shown for reference. The sequence of Env from strain HXB2 (NCBI AAB50262.1) to reference the standard numbering scheme starting from the first codon in the signal sequence of HXB2 (numbers shown in bold and underlined). Other sequences are numbered with reference to the first codon after the Aga2p signal sequence in the yeast expression constructs. Alignment was performed using Clustal Omega [86]. Asterisks indicate positions where all sequences are identical, colons indicate strong conservation, periods indicate weak conservation. Positions T605 (C605 in the QH0692dsm and YU2dsm sequences), W610, W614, L619 (Q619 in QH0692), W623, W628, W631, I635, Y638, I642, L646, W666, I682, and K683 are highlighted in red on the HXB2 sequence.
(PDF)

S2 Fig Schematic diagram of PCR reactions and plasmid recombination. (PDF)

S3 Fig Sorting gates and populations from different rounds of sorting. a) Sorting for binding to the 4E10 UCA; Representative stringent and relaxed sorting gates are shown. b) Sorting for binding to the 10E8 UCA. The indicated gates are representative of the gates used for sorting, but have been maintained the same in the different panels to show changes in the populations. Lower panel shows day-to-day variation in sorting in the distribution of the un-mutagenized cell populations. Blue dots indicates cells that have not been subjected to mutagenesis, red indicates mutagenized cells.
(PDF)

S4 Fig Representative examples of anti-V5 antibody binding to sorted clones. a) 4E10 UCA clones and b) 10E8 UCA library clones.
(PDF)

S5 Fig Binding of the mature Abs to clones from screen for 4E10 UCA-binding variants. a) binding of anti-MPER antibody 4E10; b) binding of anti-CD4 binding site antibody VRC01; c) binding of anti V3 loop antibody 447-52D; d) binding of anti-MPER antibody 4E10; e) binding of anti-MPER antibody 2F5; f) binding of anti-MPER antibody Z13e1.
(PDF)

S6 Fig Competition of MPER peptide and antibody binding. a) MPER peptide competition of mature 4E10 and 4E10 UCA binding to unmutagenized QH0692dsm reconstructed clone QH-17 containing mutations C605R W631R I642N. b) MPER peptide competition of mature 4E10 and 4E10 UCA binding to unmutagenized QH0692dsm and reconstructed clone P6-8 containing mutations L545H, L566Q, C605R, A612T, and W623R; c) MPER competition of 10E8 UCA binding to library clone C38 containing mutations K500E, K508N, Q543L, S546P, D624V, N651H, N656K, W666R, and I682F. d) MPER competition of mature 10E8 binding to unmutagenized YU2dsm and the C38 variant.
(PDF)

S7 Fig Effects of endoglycosidase H treatment. a) Effects of endoglycosidase H treatment of QH0692dsm and the reconstructed QH-17 clone containing substitutions C605R W631R I642N on binding of the indicated mature and UCA antibodies. b) Effects of endoglycosidase H treatment of YU2dsm and the reconstructed C38 clone containing substitutions K500E, K508N, Q543L, S546P, D624V, N651H, N656K, W666R, and I682F on binding of the indicated mature and UCA antibodies “Buffer” refers to samples incubated in the buffer used for endoH digestions but with no enzyme. “Isotype” refers to the isotype control antibody DAC [36].
(PDF)

S8 Fig Binding of mature Abs to clones from screen for 10E8 UCA-binding variants. a) anti-MPER antibody 4E10; b) anti-CD4 binding site antibody VRC01; c) anti V3 loop antibody 447-52D; d) anti-MPER antibody 10E8; e) anti-MPER antibody 2F5; f) anti-MPER antibody Z13e1.
(PDF)

S9 Fig Evaluation of effects of mutations in the context of different Env proteins. The identified amino acid substitutions improve anti-MPER UCA binding when transferred between corresponding positions in Env lacking stabilizing mutations and derived from different viral strains a) W666R, the predominant mutation conferring 10E8 UCA binding in the YU2dsm background also confers improved 10E8 UCA binding to a non-stabilized form of Env from viral strain YU2. b) The substitution W666R also confers improved 10E8 UCA binding in a stabilized form of Env from viral strain BG505 (BG505dsm [33]). c) The substitution W666R also confers improved 10E8 UCA binding in the context of the QH0692dsm form of Env. Note that the normal form of QH0692dsm used in this work did not contain K683 (see S1 Fig), so this was added at the C-terminal of gp41 as indicated for the purposes of making this comparison. Also, the fluorescence intensity values are low in this experiment because, as pointed out above, even the mature form of 10E8 does not bind effectively to QH0692-derived forms of Env. d) The mutations C605R, W631R, and I642N provide enhanced binding to the 4E10 UCA in the context of the YU2dsm form of Env, and not just the QH0692 form that was used to identify these mutations. As noted previously [33], there is significant binding of the 4E10 UCA to even the un-mutagenized form of YU2dsm Env. Dissociation constants and relative Bmax values for the three forms of Env are indicated on the panel.
(PDF)

S10 Fig Immunoblot of Env constructs eluted from the yeast surface yeast by reduction of disulfides. The blot was probed with anti-V5 epitope antibodies. The positions of expected migration of gp140-Aga2p, gp41-Aga2p, and Aga2p alone are indicated, as well as the migration of molecular molecular weight markers (in kDa). Cleavage at the dibasic site created by the W666R mutations would be expected to lead to enhanced density of an Aga2p-like fragment in the mutants C38 and C7, compared to the un-mutagenized YU2dsm (compare lanes 6 and 7 with lane 5). The blots indicate that under the conditions used, cleavage at the normal furin site between gp120 and gp41 is incomplete and variable among the different strains and variants.
(PDF)

S11 Fig Lack of binding of isotype control anti-interleukin 2 receptor CD25 antibody DAC to yeast-displayed Env. a) Binding of the DAC antibody (300 nM) to QH0692 gp140dsm and variants exhibiting enhanced binding to 4E10 UCA. Simultaneously assayed binding of the same strains to the 4E10 UCA (100 nM) is shown for reference. B) Representative experiment assaying binding of the DAC antibody (300 nM) to YU2 gp140dsm and variants exhibiting enhanced binding to 10E8 UCA. Simultaneously assayed binding of the same strains to the 10E8 UCA (100 nM) is shown for reference. Negative values of fluorescence intensity arise in subtracting the fluorescence of control samples incubated with fluorescent secondary antibodies alone.
(PDF)

S12 Fig Histogram showing the mutation frequencies of unselected libraries. a) QH0692 library. Position 1 on the histogram corresponds to the third nucleotide in the codon for residue Q540. b) YU2 library. Position1 on the histogram corresponds to the first nucleotide of the codon for residue S523.
(PDF)

S1 Table Individual mutant alleles recovered in screen for binding to 4E10 UCA. (PDF)

S2 Table Individual mutant alleles recovered in screen for binding to 10E8 UCA. (PDF)

S3 Table Sequence variation at sites of individual amino acid substitutions enhancing ant-MPER UCA binding. (PDF)
