# Supplementary material for: Identification of variant HIV envelope proteins with enhanced affinities for precursors to anti-gp41 broadly neutralizing antibodies
Source: PLoS One. 2019 Sep 10;14(9):e0221550. doi: 10.1371/journal.pone.0221550 (PMC6736307; doi:10.1371/journal.pone.0221550)
Supplement: S1 Fig — N-terminal signal sequences have been removed, since these were replaced by the signal sequence from Aga2p. The hydrophilic fusion peptide sequence (highlighted in green) and the optimized Kex2p-cleavage site (highlighted in purple) are as described [31]. Additional “stabilizing” mutations as described by Grimm et al., are highlighted in cyan. “SOSIP” mutations [85] are highlighted in grey and the original BG505 SOSIP sequence is shown for reference. The sequence of Env from strain HXB2 (NCBI AAB50262.1) to reference the standard numbering scheme starting from the first codon in the signal sequence of HXB2 (numbers shown in bold and underlined). Other sequences are numbered with reference to the first codon after the Aga2p signal sequence in the yeast expression constructs. Alignment was performed using Clustal Omega [86]. Asterisks indicate positions where all sequences are identical, colons indicate strong conservation, periods indicate weak conservation. Positions T605 (C605 in the QH0692dsm and YU2dsm sequences), W610, W614, L619 (Q619 in QH0692), W623, W628, W631, I635, Y638, I642, L646, W666, I682, and K683 are highlighted in red on the HXB2 sequence. (PDF) [file pone.0221550.s001.pdf]

S1 Figure.

```
YU2*          AEQLWVTVYYGVPVWKEATTTLFCASDAKAYDTEVHNVWATHACVPTDPNPQEVKLENVT 60
YU2dsm        AEQLWVTVYYGVPVWKEATTTLFCASDAKAYDTEVQNVWATHACVPTDPNPQEVKLENVT 60
BG505*        AENLWVTVYYGVPVWKDAETTLFCASDAKAYETEKHNVWATHACVPTDPNPQEIHLENVT 60
BG505SOSIP    AENLWVTVYYGVPVWKDAETTLFCASDAKAYETEKHNVWATHACVPTDPNPQEIHLENVT 60
BG505dsm      AENLWVTVYYGVPVWKDAETTLFCASDAKAYETEKQNVWATHACVPTDPNPQEIHLENVT 60
QH0692*       AENLWVTVYYGVPVWKEATTTLFCASDAKAYETEKHNVWATHACVPTDPNPQEVVLGNVT 60
QH0692dsm     AENLWVTVYYGVPVWKEATTTLFCASDAKAYETEKQNVWATHACVPTDPNPQEVVLGNVT 60
HXB2          TEKLWVTVYYGVPVWKEATTTLFCASDAKAYDTEVHNVWATHACVPTDPNPQEVVLVNVT 90
                 *************:* ************:** :*****************: * ***

YU2*          ENFNMWKNNMVEQMHEDIISLWDQSLKPCVKLTPLCVTLNCTDLRNATNTTSSS-----W 115
YU2dsm        ENFNMWKNNMVEQMHEDIISLRDQSLKPCVKLTPLCVTLNCTDLRNATNTTSSS-----W 115
BG505*        EEFNMWKNNMVEQMHTDIISLWDQSLKPCVKLTPLCVTLQCTNVTNNI------------ 108
BG505SOSIP    EEFNMWKNNMVEQMHTDIISLWDQSLKPCVKLTPLCVTLQCTNVTNNI------------ 108
BG505dsm      EEFNMWKNNMVEQMHTDIISLRDQSLKPCVKLTPLCVTLQCTNVTNNI------------ 108
QH0692*       ENFNMWKNNMVEQMHEDIISLWDESLKPCVKLTPLCVTLNCTDEVKTSYANKTSNETYKT 120
QH0692dsm     ENFNMWKNNMVEQMHEDIISLRDESLKPCVKLTPLCVTLNCTDEVKTSYANKTSNETYKT 120
HXB2          ENFNMWKNDMVEQMHEDIISLWDQSLKPCVKLTPLCVSLKCTDLKNDTNTNSSSG----R 146
              *.******:****** ***** *:*************:*:**:

YU2*          ETMEKGEIKNCSFNITTSIRDKVQKEYALFYNLDVVPIDN------------ASYRLISC 163
YU2dsm        ETMEKGEIRICSFNITTSIRDKVRKEYALFYNLNVVPIDN------------ASHRLISC 163
BG505*        TDDMRGELKNCSFNMTTELRDKKQKVYSLFYRLDVVQINENQGNRS--NNSNKEYRLINC 166
BG505SOSIP    TDDMRGELKNCSFNMTTELRDKKQKVYSLFYRLDVVQINENQGNRS--NNSNKEYRLINC 166
BG505dsm      TDDMRGELRICSFNMTTELRDKKRKVYSLFYRLNVVQINENQGNRS--NNSNKEHRLINC 166
QH0692*       SNETFGEIKNCSFSVPTGIKDKVQNVYALFYKLDVIPIDDNNNSSKNNNGSYSSYRLINC 180
QH0692dsm     SNETFGEIRICSFSVPTGIKDKVRNVYALFYKLNVIPIDDNNNSSKNNNGSYSSHRLINC 180
HXB2          MIMEKGEIKNCSFNISTSIRGKVQKEYAFFYKLDIIPIDNDT----------TSYKLTSC 196
                   **:: ***.: * ::.* :: *::**.*::: *::             .::* .*

YU2*          NTSVITQACPKVSFEPIPIHYCAPAGFAILKCNDKKFNGTGPCTNVSTVQCTHGIRPVVS 223
YU2dsm        NTSVITQACPKVSFEPIPIHYCAPAGFAILKCNDKKFNGTGPCTNVSTVQCTHGIRPVVS 223
BG505*        NTSAITQACPKVSFEPIPIHYCAPAGFAILKCKDKKFNGTGPCPSVSTVQCTHGIKPVVS 226
BG505SOSIP    NTSAITQACPKVSFEPIPIHYCAPAGFAILKCKDKKFNGTGPCPSVSTVQCTHGIKPVVS 226
BG505dsm      NTSAITQACPKVSFEPIPIHYCAPAGFAILKCKDKKFNGTGPCPSVSTVQCTHGIKPVVS 226
QH0692*       NTSVITQACPKVSFEPIPIHYCAPAGFAILKCNNKTFNGTGPCTNVSTVQCTHGIRPVVS 240
QH0692dsm     NTSVITQACPKVSFEPIPIHYCAPAGFAILKCNNKTFNGTGPCTNVSTVQCTHGIRPVVS 240
HXB2          NTSVITQACPKVSFEPIPIHYCAPAGFAILKCNNKTFNGTGPCTNVSTVQCTHGIRPVVS 256
              ***.****************************::*.******* .**********:****

YU2*          TQLLLNGSLAEEEIVIRSENFTNNAKTIIVQLNESVVINCTRPNNNTRKSIN--IGPGRA 281
YU2dsm        TQLLLNGSLAEEEIVIRSENFTNNAKTIIVQLNESVVINCTRPNNNTRKSIN--IGPGRA 281
BG505*        TQLLLNGSLAEEEVMIRSENITNNAKNILVQFNTPVQINCTRPNNNTRKSIR--IGPGQA 284
BG505SOSIP    TQLLLNGSLAEEEVMIRSENITNNAKNILVQFNTPVQINCTRPNNNTRKSIR--IGPGQA 284
BG505dsm      TQLLLNGSLAEEEVMIRSENITNNAKNILVQFNTPVQINCTRPNNNTRKSIR--IGPGQA 284
QH0692*       TQLLLNGSLAEEEVVIRSENFTNNAKTIIVHLKKSVEINCTRPGNNTRKSIH--IGPGRA 298
QH0692dsm     TQLLLNGSLAEEEVVIRSENFTNNAKTIIVHLKKSVEINCTRPGNNTRKSIH--IGPGRA 298
HXB2          TQLLLNGSLAEEEVVIRSVNFTDNAKTIIVQLNTSVEINCTRPNNNTRKRIRIQRGPGRA 316
              *************::*** *:*:***.*:*:::  * ******.***** *.   ***:*
```

```
YU2*          LYTTGEIIGDIRQAHCNLSKTQWENTLEQIAIKLKEQFGNNKTIIFNPSSGGDPEIVTHS 341
YU2dsm        LYTTGEIIGDIRQAHCNLSKTQWENTLEQIAIKLKEQYGNNKTIIFNPSSGGDPEIVTHS 341
BG505*        FYATGDIIGDIRQAHCTVSKATWNETLGKVVKQLRKHFGNNTIIRFANSSGGDLEVTTHS 344
BG505SOSIP    FYATGDIIGDIRQAHCNVSKATWNETLGKVVKQLRKHFGNNTIIRFANSSGGDLEVTTHS 344
BG505dsm      FYATGDIIGDIRQAHCNVSKATWNETLGKVVKQLRKHYGNNTIIRFANSSGGDLEVTTHS 344
QH0692*       FYATGDIIGDIRQAHCNLSSVQWNDTLKQIVIKLGEQFGTNKTIAFNQSSGGDPEIVMHS 358
QH0692dsm     FYATGDIIGDIRQAHCNLSSVQWNDTLKQIVIKLGEQYGTNKTIAFNQSSGGDPEIVMHS 358
HXB2          FVTIGK-IGNMRQAHCNISRAKWNNTLKQIASKLREQFGNNKTIIFKQSSGGDPEIVTHS 375
              : : *. **::*****.:* . *::** ::. :* :::*.*. * *  ***** *:. **

YU2*          FNCGGEFFYCNSTQLFT--WNDT-----RKLN-NTGRNITLPCRIKQIINMWQEVGKAMY 393
YU2dsm        FNCGGEFFYCNSTQLFT--WNDT-----RKLN-NTGRNITLPCRIKQINNMWQEVGKAMY 393
BG505*        FNCGGEFFYCNTSGLFNSTWISNTSV-QGSNSTGSNDSITLPCRIKQIINMWQRIGQAMY 403
BG505SOSIP    FNCGGEFFYCNTSGLFNSTWISNTSV-QGSNSTGSNDSITLPCRIKQIINMWQRIGQAMY 403
BG505dsm      FNCGGEFFYCNTSGLFNSTWISNTSV-QGSNSTGSNDSITLPCRIKQINNMWQRIGQAMY 403
QH0692*       FNCGGEFFYCNTTQLFNSTWEFHGNWTRSNFTESNSTTITLPCRIKQIVNMWQEVGKAMY 418
QH0692dsm     FNCGGEFFYCNTTQLFNSTWEFHGNWTRSNFTESNSTTITLPCRIKQINNMWQEVGKAMY 418
HXB2          FNCGGEFFYCNSTQLFNSTWFNSTWSTEGSNNTEGSDTITLPCRIKQIINMWQKVGKAMY 435
              ***********:: **.  *          . .   . .********** ****.:*:***

YU2*          APPIRGQIRCSSNITGLLLTRDGGKDTNGTEIFRPGGGDMRDNWRSELYKYKVVKIEPLG 453
YU2dsm        APPIRGQIRCSSNITGLLLTRDGGKDTNGTEIFRPGGGDMRDNWRSELYKYKVVKIEPLG 453
BG505*        APPIQGVIRCVSNITGLILTRDGGSTNSTTETFRPGGGDMRDNWRSELYKYKVVKIEPLG 463
BG505SOSIP    APPIQGVIRCVSNITGLILTRDGGSTNSTTETFRPGGGDMRDNWRSELYKYKVVKIEPLG 463
BG505dsm      APPIQGVIRCVSNITGLILTRDGGSTNSTTETFRPGGGDMRDNWRSELYKYKVVKIEPLG 463
QH0692*       APPIRGQIRCSSNITGLLLTRDGGVNG-TRETFRPGGGDMRDNWRSELYKYKVVKIEPLG 477
QH0692dsm     APPIRGQIRCSSNITGLLLTRDGGVNG-TRETFRPGGGDMRDNWRSELYKYKVVKIEPLG 477
HXB2          APPISGQIRCSSNITGLLLTRDGGNSNNESEIFRPGGGDMRDNWRSELYKYKVVKIEPLG 495
              **** * *** ******:******      * ****************************

YU2*          VAPTKAKRRVVQREK--RAVGLGALFLGFLGAAGSTMGAASITLTVQARQLLSGIVQQQN 511
YU2dsm        VAPTKCQRRVVQKREAEAATSTGATFSGFSGSAGSTMGATSITLTVQARQLLSGIVQQQN 513
BG505*        VAPTRAKRRVVGRRRRRRAVGIGAVFLGFLGAAGSTMGAASMTLTVQARNLLSGIVQQQS 523
BG505SOSIP    VAPTRCKRRVVGRRRRRRAVGIGAVFLGFLGAAGSTMGAASMTLTVQARNLLSGIVQQQS 523
BG505dsm      VAPTRCQRRVVGKREAEAATSTGATFSGFSGSAGSTMGATSMTLTVQARNLLSGIVQQQS 523
QH0692*       VAPTKAQRRVVQKREAEAATSTGATFSGFSGSAGSTMGATSITLTVQARQLLSGIVQQQN 537
QH0692dsm     VAPTKCQRRVVQKREAEAATSTGATFSGFSGSAGSTMGATSITLTVQARQLLSGIVQQQN 537
HXB2          VAPTKAKRRVVQREK--RAVGIGALFLGFLGAAGSTMGAASMTLTVQARQLLSGIVQQQN 553
              ****:.:**** :..    ..  ** * ** *:*******:*:******* *********.

YU2*          NLLRAIEAQQHLLQLTVWGIKQLQARVLAVERYLRDQQLLGIWGCSGKLICTTTVPWNTS 571
YU2dsm        NLLRAPEAQQHLLQLTVWGIKQLQARVLAVERYLRDQQLLGIWGCSGKLICCTTVPWNTS 573
BG505*        NLLRAIEAQQHLLKLTVWGIKQLQARVLAVERYLRDQQLLGIWGCSGKLICTTNVPWNSS 583
BG505SOSIP    NLLRAPEAQQHLLKLTVWGIKQLQARVLAVERYLRDQQLLGIWGCSGKLICCTNVPWNSS 583
BG505dsm      NLLRAPEAQQHLLKLTVWGIKQLQARVLAVERYLRDQQLLGIWGCSGKLICCTNVPWNSS 583
QH0692*       NLLRAIEAQQHMLQLTVWGIKQLQARVLAVERYLRDQQLLGIWGCSGKLICTTAVPWNAS 597
QH0692dsm     NLLRAPEAQQHMLQLTVWGIKQLQARVLAVERYLRDQQLLGIWGCSGKLICCTAVPWNAS 597
HXB2          NLLRAIEAQQHLLQLTVWGIKQLQARILAVERYLKDQQLLGIWGCSGKLICTTAVPWNAS 613
              ***** *****:*:************:******* *************** * ****:*
```

```
YU2*            WSNKSLNEIWDNMTWMKWEREIDNYTHIIYSLIEQSQNQQEKNEQELLALDKWASLWNWF 631
YU2dsm          WSNKSLNEIWDNMTWMKWEREIDNYTHIIYSLIEQSQNQQEKNEQELLALDKWASLWNWF 633
BG505*          WSNRNLSEIWDNMTWLQWDKEISNYTQIIYGLLEESQNQQEKNEQDLLALDKWASLWNWF 643
BG505SOSIP      WSNRNLSEIWDNMTWLQWDKEISNYTQIIYGLLEESQNQQEKNEQDLLALDKWASLWNWF 643
BG505dsm        WSNRNLSEIWDNMTWLQWDKEISNYTQIIYGLLEESQNQQEKNEQDLLALDKWASLWNWF 643
QH0692*         WSNKSQDYIWNNMTWMQWDKEINNYTNLIYSLLEDSQNQQEKNEHELLELDKWASLWNWF 657
QH0692dsm       WSNKSQDYIWNNMTWMQWDKEINNYTNLIYSLLEDSQNQQEKNEHELLELDKWASLWNWF 657
HXB2            WSNKSLEQIWNHTTWMEWDREINNYTSLIHSLIEESQNQQEKNEQELLELDKWASLWNWF 673
                ***:. . **:: **::*::**.***  *: *:*:*********::** ***********

YU2*            DITKWLWYIK   641
YU2dsm          DITKWLWYIK   643
BG505*          DISNWLWYIK     653
BG505SOSIP      DISNWLWYIK   653
BG505dsm        DISNWLWYIK   653
QH0692*         DITRWLWYI    666
QH0692dsm       DITRWLWYI    666
HXB2            NITNWLWYIK   683
                :*:.****
```

S1 Fig. Alignment of Env amino acid sequences used in this work. N-terminal signal sequences have been removed, since these were replaced by the signal sequence from Aga2p. The hydrophilic fusion peptide sequence (highlighted in green) and the optimized Kex2p-cleavage site (highlighted in purple) are as described [1]. Additional "stabilizing" mutations as described by Grimm et al., are highlighted in cyan. "SOSIP" mutations [2] are highlighted in grey and the original BG505 SOSIP sequence is shown for reference. The sequence of Env from strain HXB2 (NCBI AAB50262.1) to reference the standard numbering scheme starting from the first codon in the signal sequence of HXB2 (numbers shown in bold and underlined). Other sequences are numbered with reference to the first codon after the Aga2p signal sequence in the yeast expression constructs. Alignment was performed using Clustal Omega [3]. Asterisks indicate positions where all sequences are identical, colons indicate strong conservation, periods indicate weak conservation. Positions T605 (C605 in the QH0692dsm and YU2dsm sequences), W610, W614, L619 (Q619 in QH0692), W623, W628, W631, I635, Y638, I642, L646, W666, I682, and K683 are highlighted in red on the HXB2 sequence.

References for S1 Figure.

1. Grimm SK, Battles MB, Ackerman ME. Directed evolution of a yeast-displayed HIV-1 SOSIP gp140 spike protein toward improved expression and affinity for conformational antibodies. PLoS One. 2015;10(2):e0117227.
2. Schulke N, Vesanen MS, Sanders RW, Zhu P, Lu M, Anselma DJ, et al. Oligomeric and conformational properties of a proteolytically mature, disulfide-stabilized human immunodeficiency virus type 1 gp140 envelope glycoprotein. J Virol. 2002;76(15):7760-76.
3. Sievers F, Higgins DG. Clustal Omega, accurate alignment of very large numbers of sequences. Methods Mol Biol. 2014;1079:105-16.
