# Supplementary figures and images for: Identification of variant HIV envelope proteins with enhanced affinities for precursors to anti-gp41 broadly neutralizing antibodies
Source: PLoS One. 2019 Sep 10;14(9):e0221550. doi: 10.1371/journal.pone.0221550 (PMC6736307; doi:10.1371/journal.pone.0221550)

S2 Figure.

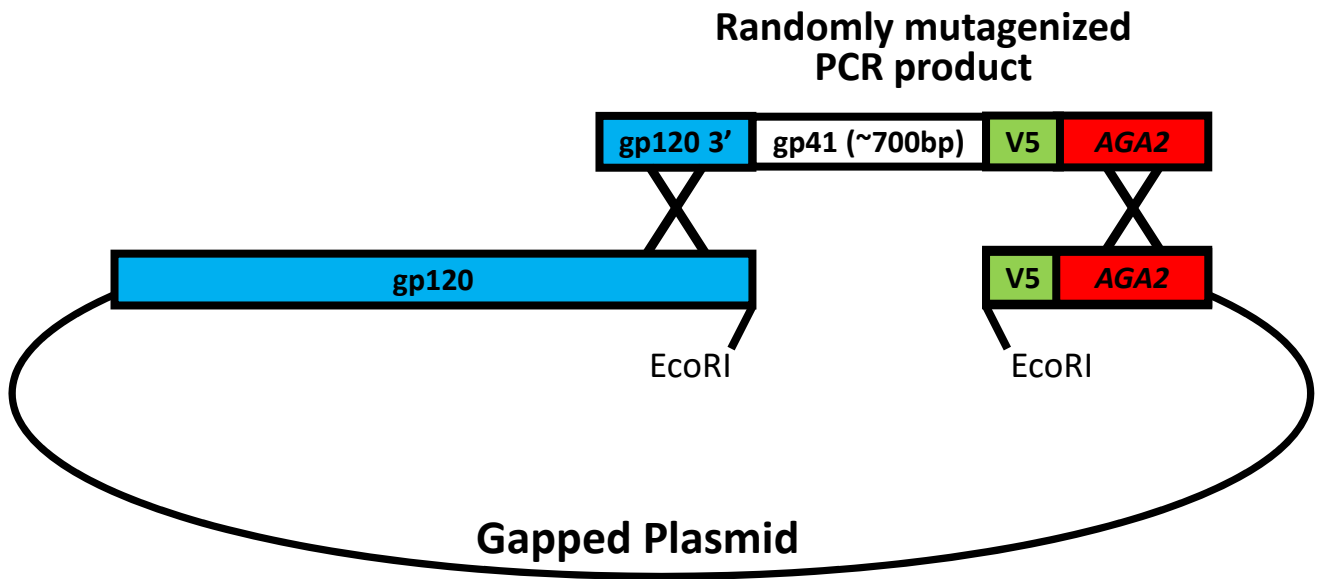

S2 Fig. Schematic diagram of PCR reactions and plasmid recombination.

Supplement: S2 Fig — (PDF) [file pone.0221550.s002.pdf]
