# Supplementary material for: Identification of variant HIV envelope proteins with enhanced affinities for precursors to anti-gp41 broadly neutralizing antibodies
Source: PLoS One. 2019 Sep 10;14(9):e0221550. doi: 10.1371/journal.pone.0221550 (PMC6736307; doi:10.1371/journal.pone.0221550)
Supplement: S3 Fig — a) Sorting for binding to the 4E10 UCA; Representative stringent and relaxed sorting gates are shown. b) Sorting for binding to the 10E8 UCA. The indicated gates are representative of the gates used for sorting, but have been maintained the same in the different panels to show changes in the populations. Lower panel shows day-to-day variation in sorting in the distribution of the un-mutagenized cell populations. Blue dots indicates cells that have not been subjected to mutagenesis, red indicates mutagenized cells. (PDF) [file pone.0221550.s003.pdf]

S3 Figure

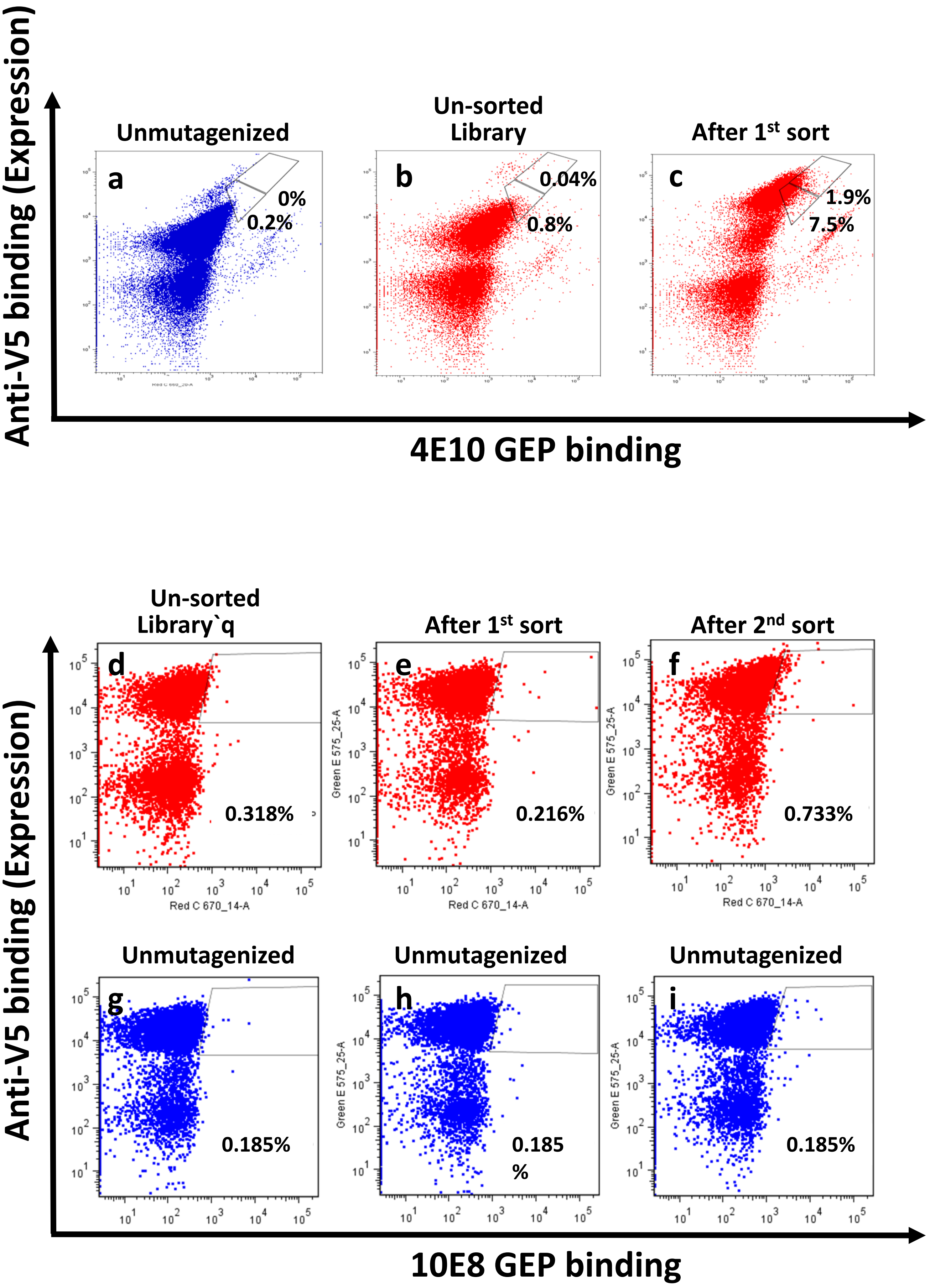

Anti-V5 binding (Expression)
Unmutagenized
Un-sorted Library
After 1st sort
a
b
c
0%
0.2%
0.04%
0.8%
1.9%
7.5%
4E10 GEP binding
Un-sorted Library`q
After 1st sort
After 2nd sort
d
e
f
0.318%
0.216%
0.733%
Red C 670_14-A
Green E 575_25-A
Unmutagenized
Unmutagenized
Unmutagenized
g
h
i
0.185%
0.185 %
0.185%
10E8 GEP binding

S3 Fig. Sorting gates and populations from different rounds of sorting. a) Sorting for binding to the 4E10 UCA; Representative stringent and relaxed sorting gates are shown. b) Sorting for binding to the 10E8 UCA. The indicated gates are representative of the gates used for sorting, but have been maintained the same in the different panels to show changes in the populations. Lower panel shows day-to-day variation in sorting in the distribution of the un-mutagenized cell populations. Blue dots indicates cells that have not been subjected to mutagenesis, red indicates mutagenized cells.
