# Supplementary material for: Identification of variant HIV envelope proteins with enhanced affinities for precursors to anti-gp41 broadly neutralizing antibodies
Source: PLoS One. 2019 Sep 10;14(9):e0221550. doi: 10.1371/journal.pone.0221550 (PMC6736307; doi:10.1371/journal.pone.0221550)
Supplement: S4 Fig — a) 4E10 UCA clones and b) 10E8 UCA library clones. (PDF) [file pone.0221550.s004.pdf]

S4 Figure

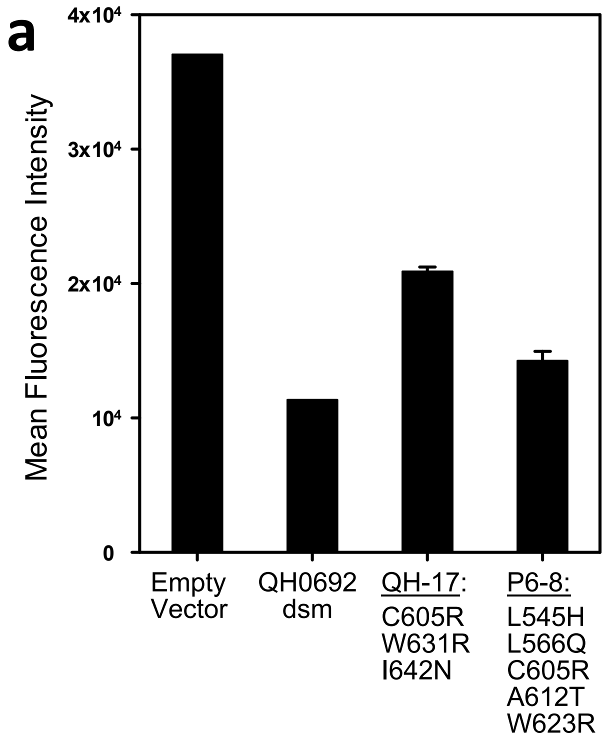

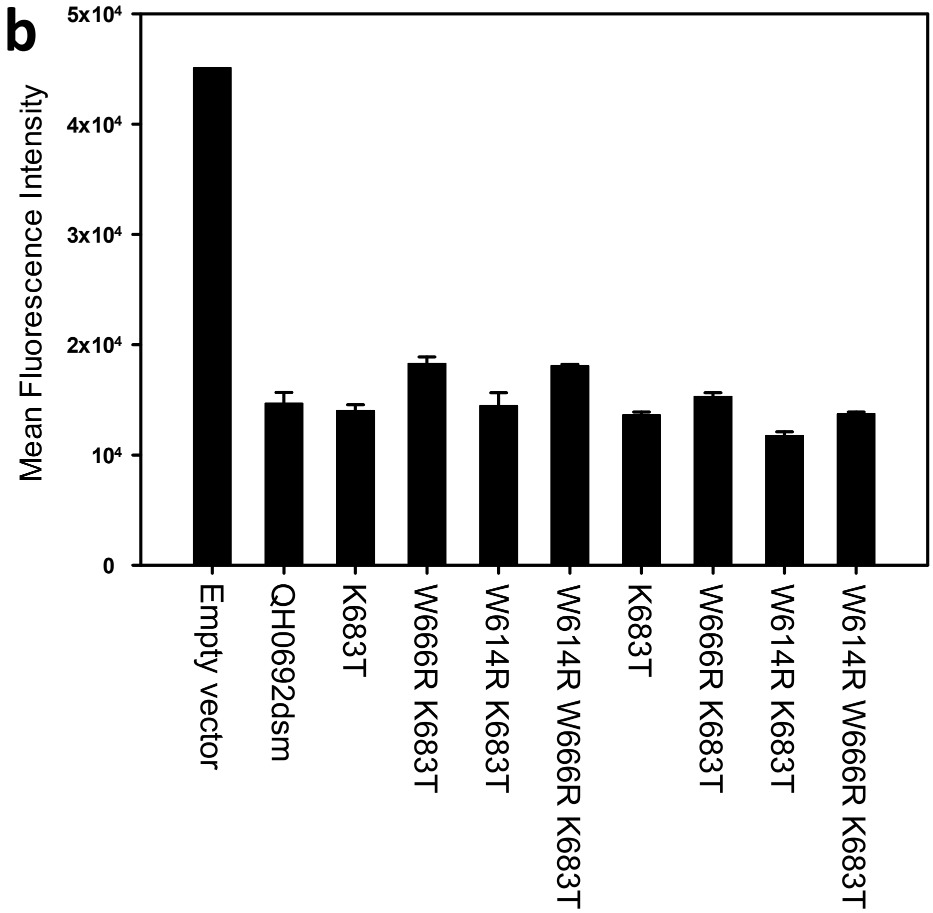

S4 Fig. Representative examples of anti-V5 antibody binding to sorted clones. a) 4E10 UCA clones and b) 10E8 UCA library clones.
