# Supplementary material for: Identification of variant HIV envelope proteins with enhanced affinities for precursors to anti-gp41 broadly neutralizing antibodies
Source: PLoS One. 2019 Sep 10;14(9):e0221550. doi: 10.1371/journal.pone.0221550 (PMC6736307; doi:10.1371/journal.pone.0221550)
Supplement: S5 Fig — a) binding of anti-MPER antibody 4E10; b) binding of anti-CD4 binding site antibody VRC01; c) binding of anti V3 loop antibody 447-52D; d) binding of anti-MPER antibody 4E10; e) binding of anti-MPER antibody 2F5; f) binding of anti-MPER antibody Z13e1. (PDF) [file pone.0221550.s005.pdf]

S5 Figure

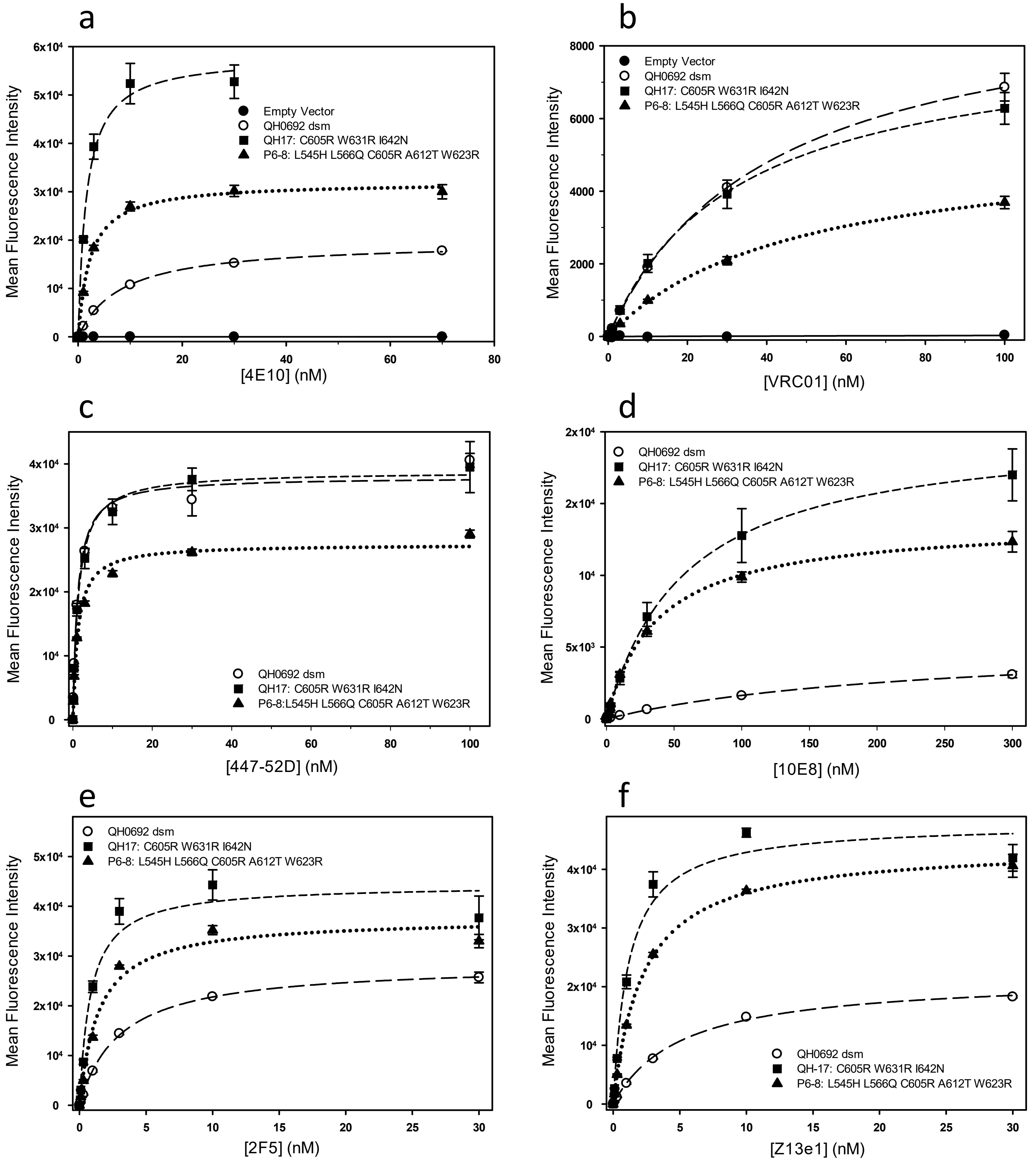
a
Mean Fluorescence Intensity
[4E10] (nM)
Empty Vector
QH0692 dsm
QH17: C605R W631R I642N
P6-8: L545H L566Q C605R A612T W623R
b
Mean Fluorescence Intensity
[VRC01] (nM)
Empty Vector
QH0692 dsm
QH17: C605R W631R I642N
P6-8: L545H L566Q C605R A612T W623R
c
Mean Fluorescence Inensity
[447-52D] (nM)
QH0692 dsm
QH17: C605R W631R I642N
P6-8:L545H L566Q C605R A612T W623R
d
Mean Fluorescence Intensity
[10E8] (nM)
QH0692 dsm
QH17: C605R W631R I642N
P6-8: L545H L566Q C605R A612T W623R
e
Mean Fluorescence Intensity
[2F5] (nM)
QH0692 dsm
QH17: C605R W631R I642N
P6-8: L545H L566Q C605R A612T W623R
f
Mean Fluorescence Intensity
[Z13e1] (nM)
QH0692 dsm
QH-17: C605R W631R I642N
P6-8: L545H L566Q C605R A612T W623R

S5 Fig. Binding of the mature Abs to clones from screen for 4E10 UCA-binding variants. a) binding of anti-MPER antibody 4E10; b) binding of anti-CD4 binding site antibody VRC01; c) binding of anti V3 loop antibody 447-52D; d) binding of anti-MPER antibody 4E10; e) binding of anti-MPER antibody 2F5; f) binding of anti-MPER antibody Z13e1.
