# Supplementary material for: Identification of variant HIV envelope proteins with enhanced affinities for precursors to anti-gp41 broadly neutralizing antibodies
Source: PLoS One. 2019 Sep 10;14(9):e0221550. doi: 10.1371/journal.pone.0221550 (PMC6736307; doi:10.1371/journal.pone.0221550)
Supplement: S6 Fig — a) MPER peptide competition of mature 4E10 and 4E10 UCA binding to unmutagenized QH0692dsm reconstructed clone QH-17 containing mutations C605R W631R I642N. b) MPER peptide competition of mature 4E10 and 4E10 UCA binding to unmutagenized QH0692dsm and reconstructed clone P6-8 containing mutations L545H, L566Q, C605R, A612T, and W623R; c) MPER competition of 10E8 UCA binding to library clone C38 containing mutations K500E, K508N, Q543L, S546P, D624V, N651H, N656K, W666R, and I682F. d) MPER competition of mature 10E8 binding to unmutagenized YU2dsm and the C38 variant. (PDF) [file pone.0221550.s006.pdf]

S6 Figure.

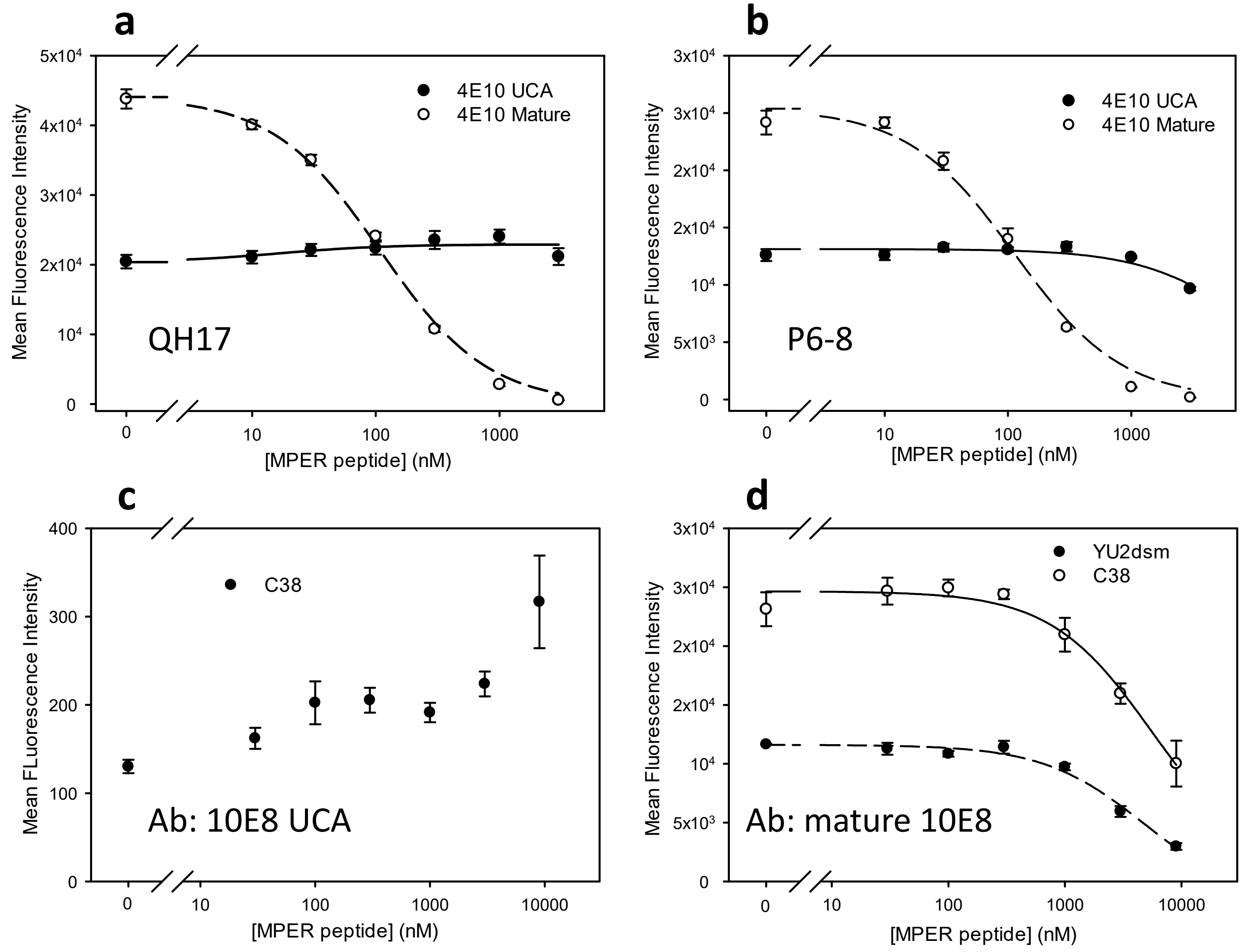

S6 Fig. Competition of MPER peptide and antibody binding. a) MPER peptide competition of mature 4E10 and 4E10 UCA binding to unmutagenized QH0692dsm reconstructed clone QH17 containing mutations C605R W631R I642N. b) MPER peptide competition of mature 4E10 and 4E10 UCA binding to unmutagenized QH0692dsm and reconstructed clone P6-8 containing mutations L545H, L566Q, C605R, A612T, and W623R; c) MPER competition of 10E8 UCA binding to library clone C38 containing mutations K500E, K508N, Q543L, S546P, D624V, N651H, N656K, W666R, and I682F. d) MPER competition of mature 10E8 binding to unmutagenized YU2dsm and the C38 variant.
