# Supplementary material for: Identification of variant HIV envelope proteins with enhanced affinities for precursors to anti-gp41 broadly neutralizing antibodies
Source: PLoS One. 2019 Sep 10;14(9):e0221550. doi: 10.1371/journal.pone.0221550 (PMC6736307; doi:10.1371/journal.pone.0221550)
Supplement: S7 Fig — a) Effects of endoglycosidase H treatment of QH0692dsm and the reconstructed QH-17 clone containing substitutions C605R W631R I642N on binding of the indicated mature and UCA antibodies. b) Effects of endoglycosidase H treatment of YU2dsm and the reconstructed C38 clone containing substitutions K500E, K508N, Q543L, S546P, D624V, N651H, N656K, W666R, and I682F on binding of the indicated mature and UCA antibodies “Buffer” refers to samples incubated in the buffer used for endoH digestions but with no enzyme. “Isotype” refers to the isotype control antibody DAC [36]. (PDF) [file pone.0221550.s007.pdf]

S7 Figure.

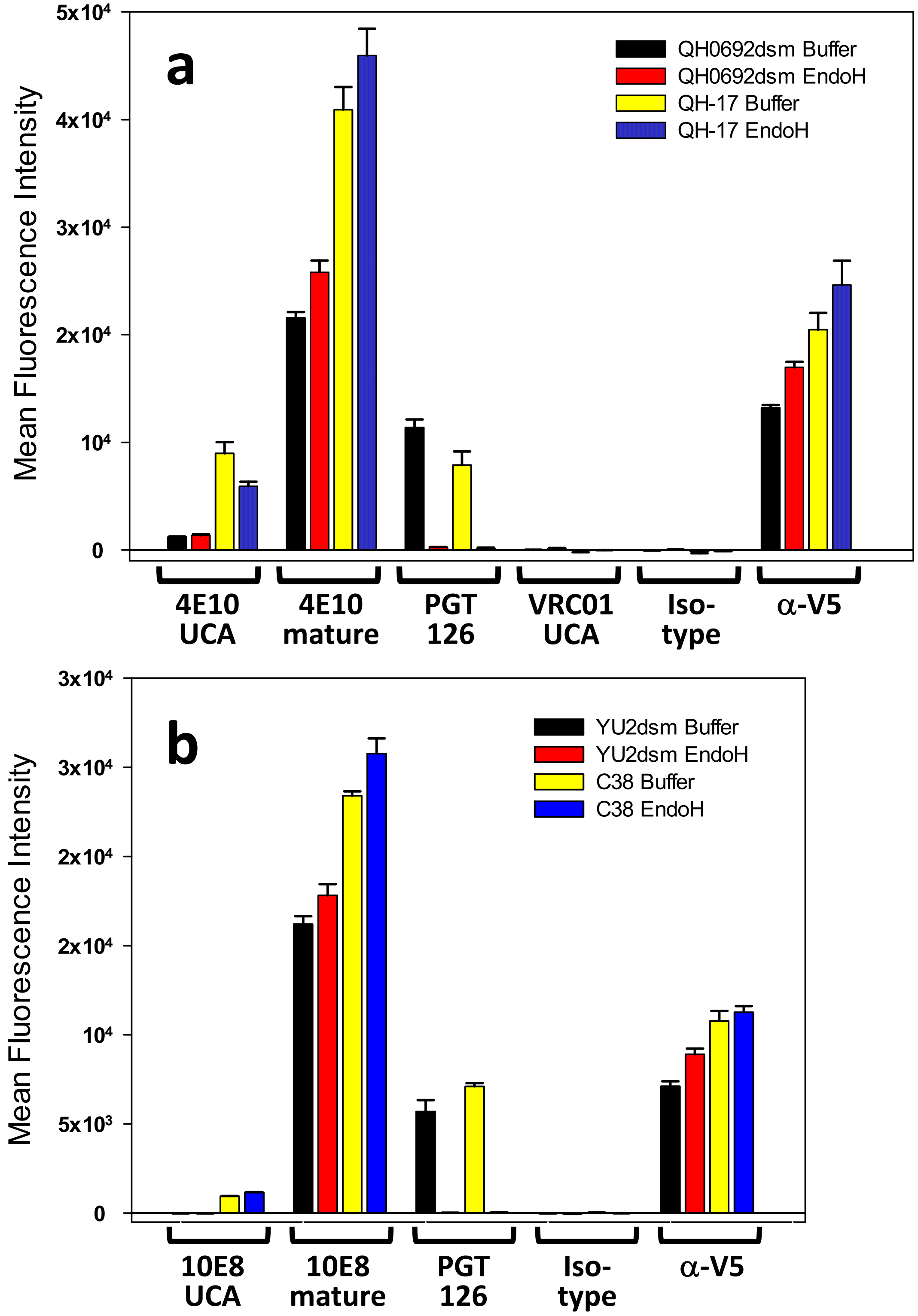

S7 Fig. Effects of endoglycosidase H treatment. a) Effects of endoglycosidase H treatment of QH0692dsm and the reconstructed QH17 clone containing substitutions C605R W631R I642N on binding of the indicated mature and UCA antibodies. b) Effects of endoglycosidase H treatment of YU2dsm and the reconstructed C38 clone containing substitutions K500E, K508N, Q543L, S546P, D624V, N651H, N656K, W666R, and I682F on binding of the indicated mature and UCA antibodies "Buffer" refers to samples incubated in the buffer used for endoH digestions but with no enzyme. "Isotype" refers to the isotype control antibody DAC {Vincenti, 1998 #407}.
