# Supplementary material for: Identification of variant HIV envelope proteins with enhanced affinities for precursors to anti-gp41 broadly neutralizing antibodies
Source: PLoS One. 2019 Sep 10;14(9):e0221550. doi: 10.1371/journal.pone.0221550 (PMC6736307; doi:10.1371/journal.pone.0221550)
Supplement: S8 Fig — a) anti-MPER antibody 4E10; b) anti-CD4 binding site antibody VRC01; c) anti V3 loop antibody 447-52D; d) anti-MPER antibody 10E8; e) anti-MPER antibody 2F5; f) anti-MPER antibody Z13e1. (PDF) [file pone.0221550.s008.pdf]

S8 Figure

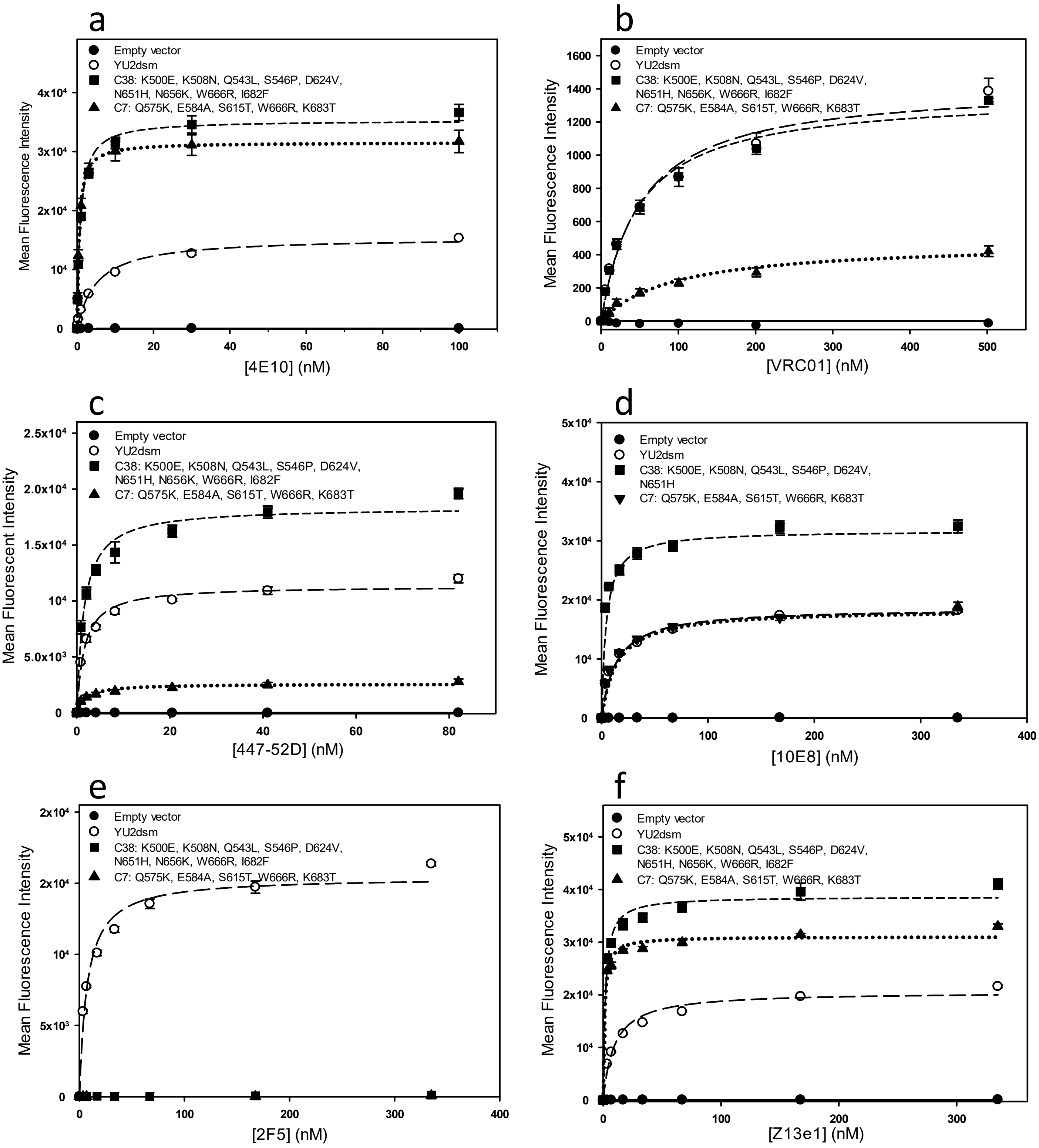

S8 Fig. Binding of mature Abs to clones from screen for 10E8 UCA-binding variants. a) anti-MPER antibody 4E10; b) anti-CD4 binding site antibody VRC01; c) anti V3 loop antibody 447-52D; d) anti-MPER antibody 10E8; e) anti-MPER antibody 2F5; f) anti-MPER antibody Z13e1.
