# Supplementary material for: Identification of variant HIV envelope proteins with enhanced affinities for precursors to anti-gp41 broadly neutralizing antibodies
Source: PLoS One. 2019 Sep 10;14(9):e0221550. doi: 10.1371/journal.pone.0221550 (PMC6736307; doi:10.1371/journal.pone.0221550)
Supplement: S9 Fig — The identified amino acid substitutions improve anti-MPER UCA binding when transferred between corresponding positions in Env lacking stabilizing mutations and derived from different viral strains a) W666R, the predominant mutation conferring 10E8 UCA binding in the YU2dsm background also confers improved 10E8 UCA binding to a non-stabilized form of Env from viral strain YU2. b) The substitution W666R also confers improved 10E8 UCA binding in a stabilized form of Env from viral strain BG505 (BG505dsm [33]). c) The substitution W666R also confers improved 10E8 UCA binding in the context of the QH0692dsm form of Env. Note that the normal form of QH0692dsm used in this work did not contain K683 (see S1 Fig), so this was added at the C-terminal of gp41 as indicated for the purposes of making this comparison. Also, the fluorescence intensity values are low in this experiment because, as pointed out above, even the mature form of 10E8 does not bind effectively to QH0692-derived forms of Env. d) The mutations C605R, W631R, and I642N provide enhanced binding to the 4E10 UCA in the context of the YU2dsm form of Env, and not just the QH0692 form that was used to identify these mutations. As noted previously [33], there is significant binding of the 4E10 UCA to even the un-mutagenized form of YU2dsm Env. Dissociation constants and relative Bmax values for the three forms of Env are indicated on the panel. (PDF) [file pone.0221550.s009.pdf]

S9 Figure.

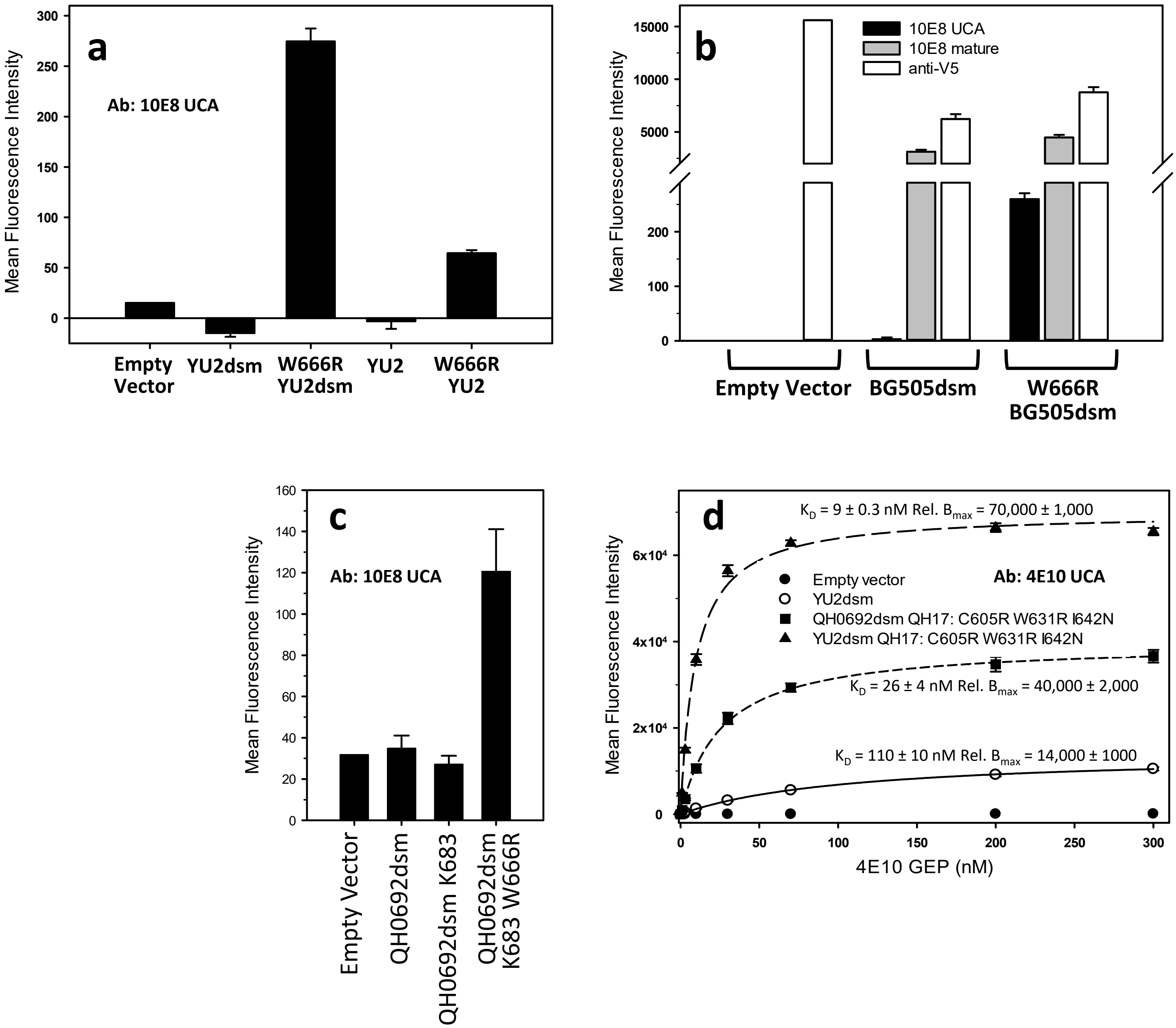

S9 Fig. Evaluation of effects of mutations in the context of different Env proteins. The identified amino acid substitutions improve anti-MPER UCA binding when transferred between corresponding positions in Env lacking stabilizing mutations and derived from different viral strains a) W666R, the predominant mutation conferring 10E8 UCA binding in the YU2dsm background also confers improved 10E8 UCA binding to a non-stabilized form of Env from viral strain YU2. b) The substitution W666R also confers improved 10E8 UCA binding in a stabilized form of Env from viral strain BG505 (BG505dsm {Mathew, 2018 #382}). c) The substitution W666R also confers improved 10E8 UCA binding in the context of the QH0692dsm form of Env. Note that the normal form of QH0692dsm used in this work did not contain K683 (see S1 Figure 1), so this was added at the C-terminal of gp41 as indicated for the purposes of making this comparison. Also, the fluorescence intensity values are low in this experiment because, as pointed out above, even the mature form of 10E8 does not bind effectively to QH0692-derived forms of Env. d) The mutations C605R, W631R, and I642N provide enhanced binding to the 4E10 UCA in the context of the YU2dsm form of Env, and not just the QH0692 form that was used to identify these mutations. As noted previously {Mathew, 2018 #382}, there is significant binding of the 4E10 UCA to even the un-mutagenized form of YU2dsm Env. Dissociation constants and relative B_{max} values for the three forms of Env are indicated on the panel.
