# Supplementary material for: Identification of variant HIV envelope proteins with enhanced affinities for precursors to anti-gp41 broadly neutralizing antibodies
Source: PLoS One. 2019 Sep 10;14(9):e0221550. doi: 10.1371/journal.pone.0221550 (PMC6736307; doi:10.1371/journal.pone.0221550)
Supplement: S10 Fig — The blot was probed with anti-V5 epitope antibodies. The positions of expected migration of gp140-Aga2p, gp41-Aga2p, and Aga2p alone are indicated, as well as the migration of molecular molecular weight markers (in kDa). Cleavage at the dibasic site created by the W666R mutations would be expected to lead to enhanced density of an Aga2p-like fragment in the mutants C38 and C7, compared to the un-mutagenized YU2dsm (compare lanes 6 and 7 with lane 5). The blots indicate that under the conditions used, cleavage at the normal furin site between gp120 and gp41 is incomplete and variable among the different strains and variants. (PDF) [file pone.0221550.s010.pdf]

S10 Figure.

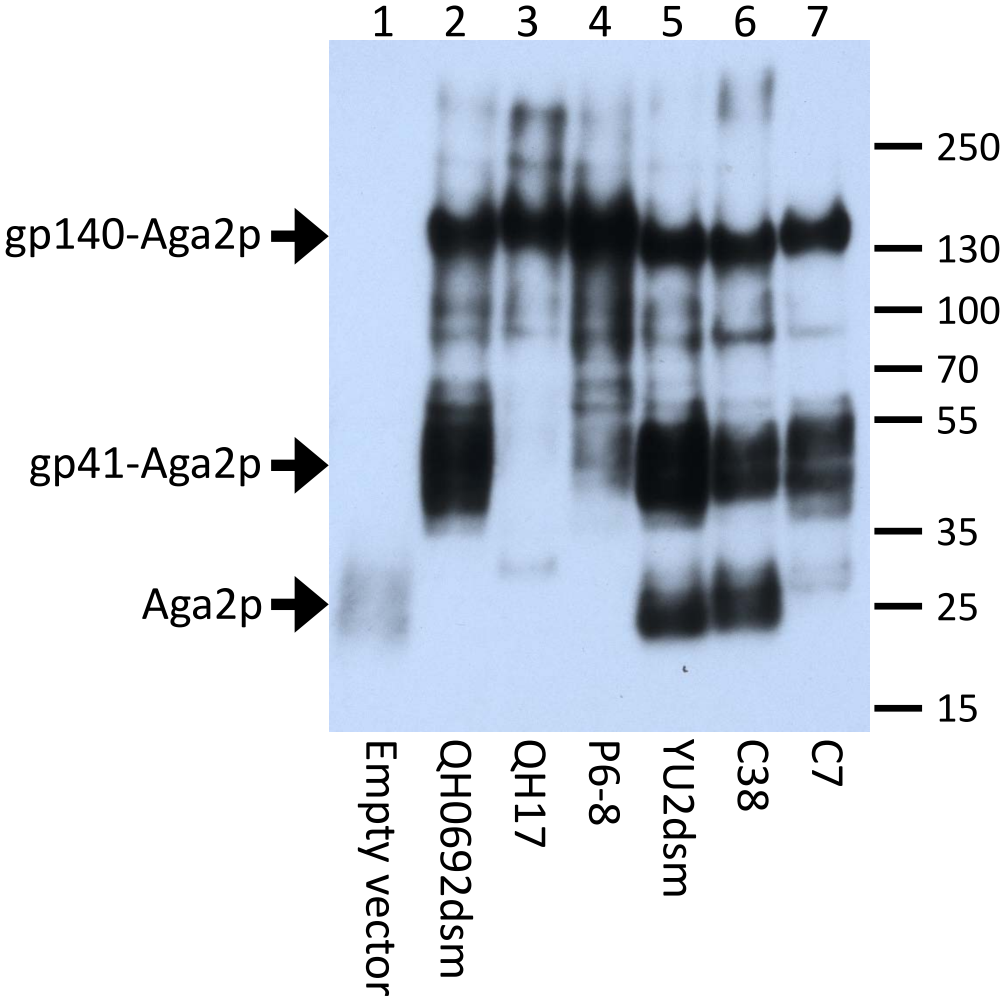

S10 Fig. Immunoblot of Env constructs eluted from the yeast surface yeast by reduction of disulfides. The blot was probed with anti-V5 epitope antibodies. The positions of expected migration of gp140-Aga2p, gp41-Aga2p, and Aga2p alone are indicated, as well as the migration of molecular molecular weight markers (in kDa). Cleavage at the dibasic site created by the W666R mutations would be expected to lead to enhanced density of an Aga2p-like fragment in the mutants C38 and C7, compared to the un-mutagenized YU2dsm (compare lanes 6 and 7 with lane 5). The blots indicate that under the conditions used, cleavage at the normal furin site between gp120 and gp41 is incomplete and variable among the different strains and variants.
