# Supplementary material for: Identification of variant HIV envelope proteins with enhanced affinities for precursors to anti-gp41 broadly neutralizing antibodies
Source: PLoS One. 2019 Sep 10;14(9):e0221550. doi: 10.1371/journal.pone.0221550 (PMC6736307; doi:10.1371/journal.pone.0221550)
Supplement: S11 Fig — a) Binding of the DAC antibody (300 nM) to QH0692 gp140dsm and variants exhibiting enhanced binding to 4E10 UCA. Simultaneously assayed binding of the same strains to the 4E10 UCA (100 nM) is shown for reference. B) Representative experiment assaying binding of the DAC antibody (300 nM) to YU2 gp140dsm and variants exhibiting enhanced binding to 10E8 UCA. Simultaneously assayed binding of the same strains to the 10E8 UCA (100 nM) is shown for reference. Negative values of fluorescence intensity arise in subtracting the fluorescence of control samples incubated with fluorescent secondary antibodies alone. (PDF) [file pone.0221550.s011.pdf]

Supplemental Figure S11

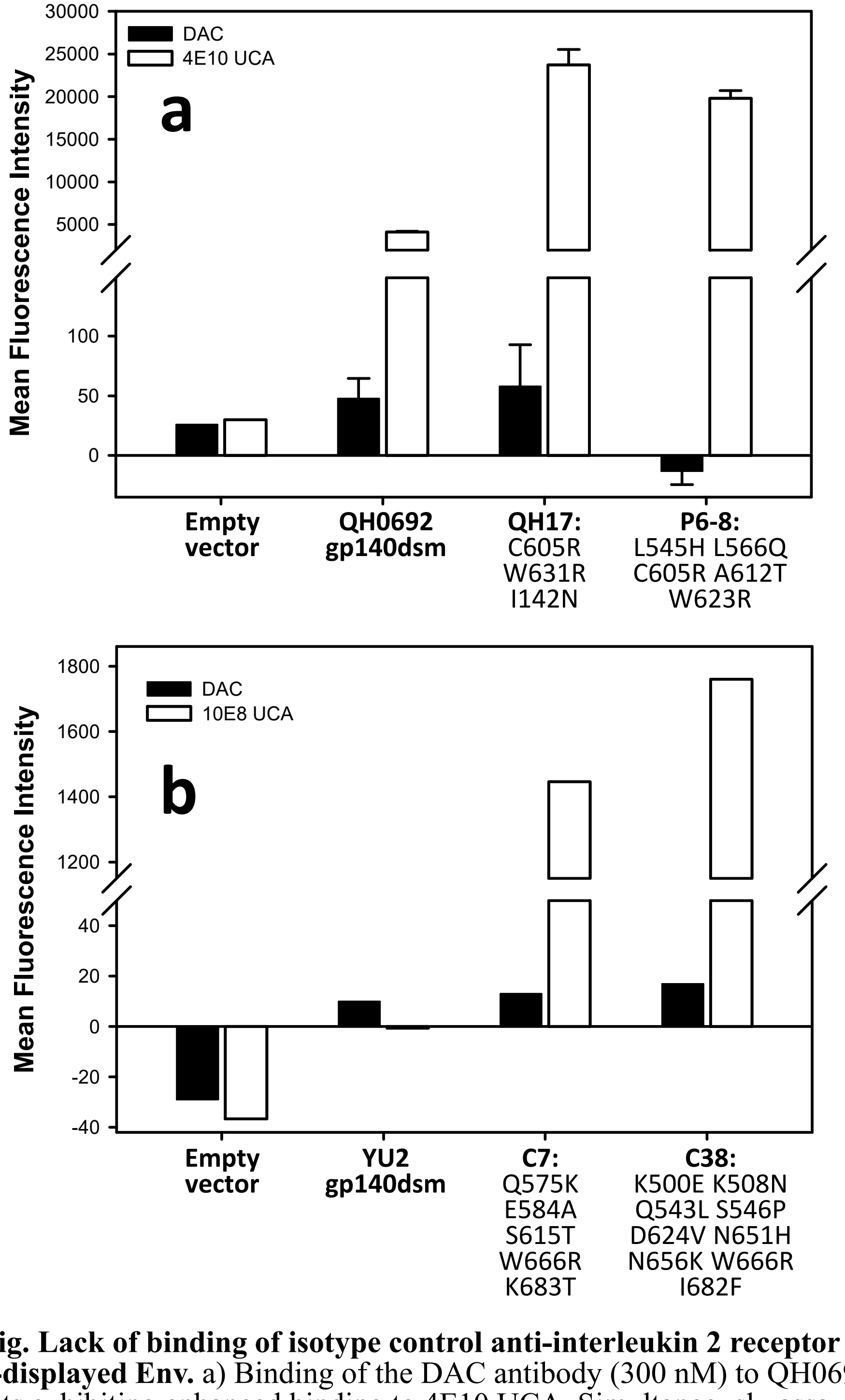

S11 Fig. Lack of binding of isotype control anti-interleukin 2 receptor CD25 antibody DAC to yeast-displayed Env. a) Binding of the DAC antibody (300 nM) to QH0692 gp140dsm and variants exhibiting enhanced binding to 4E10 UCA. Simultaneously assayed binding of the same strains to the 4E10 UCA (100 nM) is shown for reference. B) Representative experiment assaying binding of the DAC antibody (300 nM) to YU2 gp140dsm and variants exhibiting enhanced binding to 10E8 UCA. Simultaneously assayed binding of the same strains to the 10E8 UCA (100 nM) is shown for reference. Negative values of fluorescence intensity arise in subtracting the fluorescence of control samples incubated with fluorescent secondary antibodies alone.
