# Supplementary material for: Identification of variant HIV envelope proteins with enhanced affinities for precursors to anti-gp41 broadly neutralizing antibodies
Source: PLoS One. 2019 Sep 10;14(9):e0221550. doi: 10.1371/journal.pone.0221550 (PMC6736307; doi:10.1371/journal.pone.0221550)
Supplement: S12 Fig — a) QH0692 library. Position 1 on the histogram corresponds to the third nucleotide in the codon for residue Q540. b) YU2 library. Position1 on the histogram corresponds to the first nucleotide of the codon for residue S523. (PDF) [file pone.0221550.s012.pdf]

S12 Figure

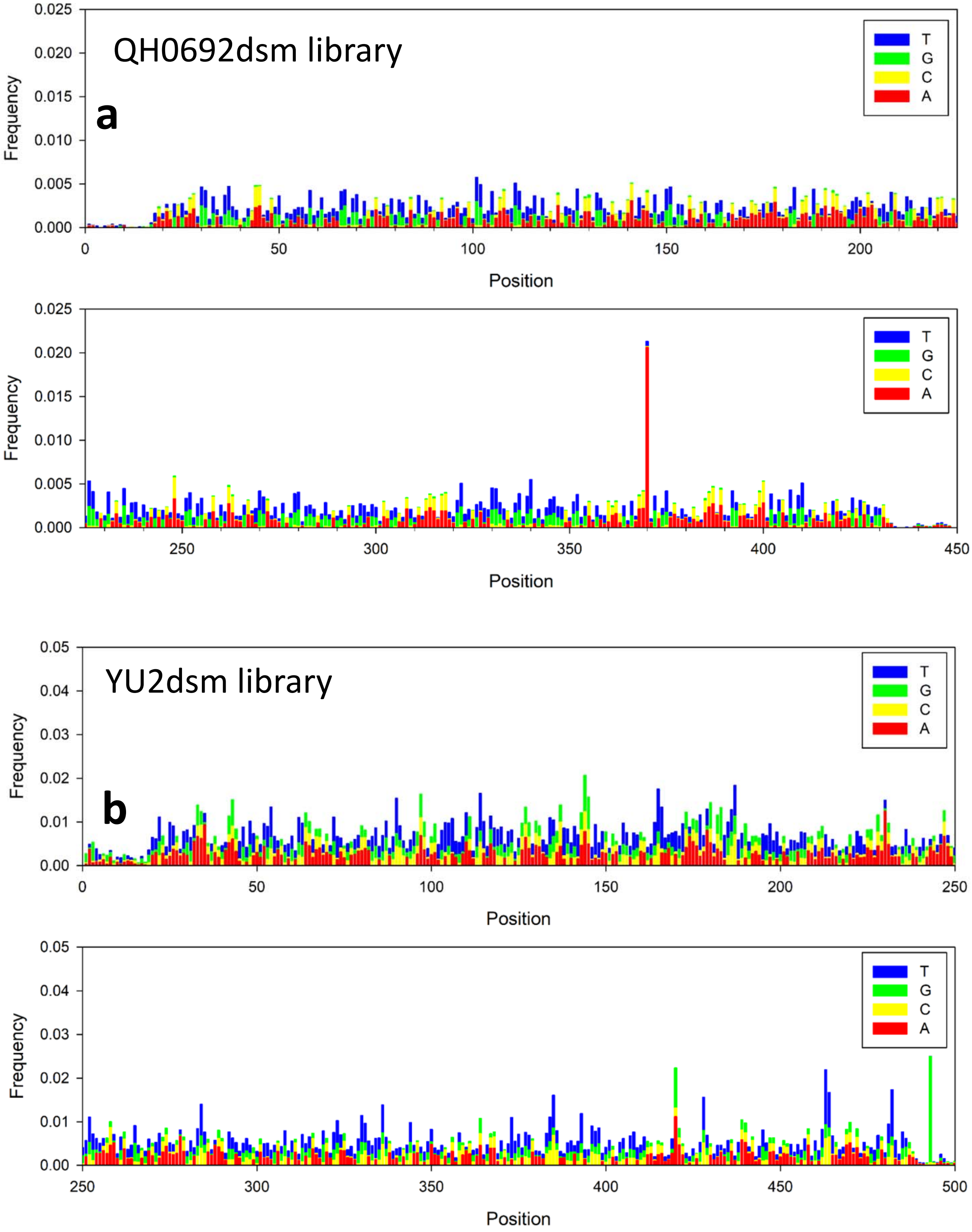

S12 Fig. Histogram showing the mutation frequencies of unselected libraries. a) QH0692 library. Position 1 on the histogram corresponds to the third nucleotide in the codon for residue Q540. b) YU2 library. Position1 on the histogram corresponds to the first nucleotide of the codon for residue S523.
