# Supplementary material for: Identification of variant HIV envelope proteins with enhanced affinities for precursors to anti-gp41 broadly neutralizing antibodies
Source: PLoS One. 2019 Sep 10;14(9):e0221550. doi: 10.1371/journal.pone.0221550 (PMC6736307; doi:10.1371/journal.pone.0221550)
Supplement: S1 Table — (PDF) [file pone.0221550.s013.pdf]

S1 Table. Individual mutant alleles recovered in screen for binding to 4E10 UCA.

Clone ID[a]	Amino acid substitutions[b]
P4-6	G459V V549A
P4-7	Q591R I603F N611S **W631R**
P4-8	G459V V506D E514D Q562H C605R
P5-1	T569S Q590H I603F W666R
P5-2	G459V V549L **W631R** L646S
P5-3	G459V T529A **W631R** E662V
P5-4	T514A E584K G600D C604S R677K
P5-5	Q653H
P6-6	G459V G572C V583D **W631R**
P6-8	G459V L545H L566Q C605R A612T W623R
P6-10	G459V W623R W628R
QH-11	T538R, Q551R, K601E, C605R, M629L, **W631R**, E634D
QH-12	G459V I595M W623R I635K
QH-13	**W631R** L663V
QH-17	G459V C605R **W631R** I642N
QH-18	**W631R** D648Y
QH-19	T515X[c] **W631R**
QH-20	S534C L593P W610R
QH-21	Q562H I635M W666R W670R
QH-25	**W631R**
P4G-1	G459V I603N S615T **W631R**
P4G-3	G459V C605R Q619P E654G
P4G-4	N616K S618P **W631R**
P4G-5	N553I G572D W623R **W631R**
P4G-6	G459V Q550H Q562H K574Q Q590K W610R **W631R**
P4G-9	G459V Q550H Q630R **W631R** N636S
P4G-10	G459V V549A Q630R **W631R**
P4G-11	L568Q L581F W623R **W631R**

[a]The listed clones exhibited greater than 2-fold increases in fluorescence compared to the unmutagenized starting form of QH0692 Env in a single point binding assay using 260 nM 4E10 UCA.

[b]The mutation G459V was found to be present in in a subpopulation of the preparation of plasmid used as a template for library generation. It does not affect antibody binding and was not included in any of the clones re-created by site direct mutagenesis. Note that mutations at positions 459 and 506 (in clone pMDP4-8) are outside the region targeted for random mutagenesis. The predominant W631R mutation is shown in bold.

[c]ambiguous read
