# Supplementary material for: Identification of variant HIV envelope proteins with enhanced affinities for precursors to anti-gp41 broadly neutralizing antibodies
Source: PLoS One. 2019 Sep 10;14(9):e0221550. doi: 10.1371/journal.pone.0221550 (PMC6736307; doi:10.1371/journal.pone.0221550)
Supplement: S2 Table — (PDF) [file pone.0221550.s014.pdf]

S2 Table. Individual mutant alleles recovered in screen for binding to 10E8 UCA.

Clone ID[a]	Amino acid substitutions[a]
C2	**W666R**[b]
C3	F522C L537M R579S C598Y **W666R**
C6	R633I **W666R**
C7	Q575K E584A S615T **W666R** K683T
C8	E510V Q567L D664E **W666R**
C9	N651I L661M **W666R** I682V
C11	T513A E632A **W666R**
C12	P498H S534A L566M W614R N637T Y638N Y643H Q648R A667S
C14	L555M W614R D664G
C16	V506D T533N V549A A558E H564L Q575H V583D E634D L661M **W666R** K677N
C17	S481A K485T G527D I548F N620I E647D **W666R** L669W
C19	C501A Q502K R503S R504S L537M N656K **W666R**
C23	I535S S546T N554I **W666R** K683R
C24	A510aS[c] S615T Q658P **W666R**
C26	S514Y **W666R**
C28	E510Q C598S **W666R**
C29	Y484N A532D K630N E657G **W666R**
C32	C501A Q502K R503S R504S N656K L660V **W666R**
C33	C501A Q502K R503S R504S L581M **W666R**
C36	A512D K601T **W666R** I682S
C37	N637T **W666R** A667S
C38	K500E K508N Q543L S546P D624V N651H N656K **W666R** I682F
C40	C501A Q502K R503S R504S **W666R**
C42	Q543R E621K E657G
C46	Q591R G600C L660M **W666R**
C47	L545V N625I **W666R** W680R K683T
C48	V549D Q551H **W666R**

[a]The listed clones exhibited greater than 2-fold increases in fluorescence compared to the unmutagenized starting form of YU2 Env in a single point binding assay using 340 nM 10E8 UCA.

[b]The predominant W666R mutation is shown in bold.

[c]Residue A510a is in a region of insertion in comparing the YU2 gp140dsm sequence with the sequence from strain HXB2 used as a reference for numbering.
