# Supplementary material for: Identification of variant HIV envelope proteins with enhanced affinities for precursors to anti-gp41 broadly neutralizing antibodies
Source: PLoS One. 2019 Sep 10;14(9):e0221550. doi: 10.1371/journal.pone.0221550 (PMC6736307; doi:10.1371/journal.pone.0221550)
Supplement: S3 Table — (PDF) [file pone.0221550.s015.pdf]

S3 Table. Sequence variation at sites of individual amino acid substitutions enhancing ant-MPER UCA binding (from the AnalyzeAlign Los Alamos National Laboratory Database; http://www.hiv.lanl.gov).

Amino acid position (HXB2 numbering)	Frequency (numbers; out of 6636 total)
C605 (QH0692) , T605 (YU2)	T: 92.51% (5061) P: 4.62% (253) other: 2.87% (157)
W610	W: 99.60% (5447) other: 0.40% (22)
W614	W: 99.60% (5444) other: 0.40% (22)
Q619	L: 33.79% (1843) Y: 22.18% (1210) Q: 21.87% (1193) E: 3.90% (213) F: 3.74% (204) H: 3.15% (172) K: 3.01% (164) V: 2.49% (136) M: 2.11% (115) other: 3.76% (205)
W623	W: 99.09% (5419) other: 0.91% (50)
W628	W: 99.76% (5458) other: 0.24% (13)
W631	W: 99.76% (5458) other: 0.24% (13)
I635	I: 94.97% (5195) V: 4.88% (267) other: 0.15% (8)
Y638	Y: 97.81% (5349) other: 2.19% (120)
I642	I: 99.63% (5449) other: 0.37% (20)
L646	L: 60.57% (3313) I: 39.20% (2144) other: 0.24% (13)
W666	W: 99.82% (5461) other: 0.18% (10)
I682	I: 99.51% (5443) other: 0.49% (27)
K683	K: 77.82% (4257) R: 21.39% (1170) other: 0.79% (43)
